# Spontaneous Water Radical Cation Oxidation at Double Bonds in Microdroplets

**DOI:** 10.3389/fchem.2022.903774

**Published:** 2022-04-26

**Authors:** Lingqi Qiu, Nicolás M. Morato, Kai-Hung Huang, R. Graham Cooks

**Affiliations:** Department of Chemistry and Center for Analytical Instrumentation Development, Purdue University, West Lafayette, IN, United States

**Keywords:** mass spectrometry, reaction acceleration, interfacial reaction, ionization, solvation, ion thermochemistry, high-throughput screening, fragmentation mechanism

## Abstract

Spontaneous oxidation of compounds containing diverse X=Y moieties (e.g., sulfonamides, ketones, esters, sulfones) occurs readily in organic-solvent microdroplets. This surprising phenomenon is proposed to be driven by the generation of an intermediate species [M+H_2_O]^+·^: a covalent adduct of water radical cation (H_2_O^**+·**^) with the reactant molecule (M). The adduct is observed in the positive ion mass spectrum while its formation in the interfacial region of the microdroplet (i.e., at the air-droplet interface) is indicated by the strong dependence of the oxidation product formation on the spray distance (which reflects the droplet size and consequently the surface-to-volume ratio) and the solvent composition. Importantly, based on the screening of a ca. 21,000-compound library and the detailed consideration of six functional groups, the formation of a molecular adduct with the water radical cation is a significant route to ionization in positive ion mode electrospray, where it is favored in those compounds with X=Y moieties which lack basic groups. A set of model monofunctional systems was studied and in one case, benzyl benzoate, evidence was found for oxidation driven by hydroxyl radical adduct formation followed by protonation in addition to the dominant water radical cation addition process. Significant implications of molecular ionization by water radical cations for oxidation processes in atmospheric aerosols, analytical mass spectrometry and small-scale synthesis are noted.

## 1 Introduction

Microdroplet chemistry is an emerging field in chemistry due to the remarkable reaction acceleration (Girod et al., 2011; Müller et al., 2012; Yan et al., 2016, 2017; Lee et al., 2017; Nam et al., 2017; Cooks and Yan, 2018; Sahota et al., 2019; Zhang et al., 2019, Zhang et al., 2021 Y.; Wei et al., 2020, 2022; Cao et al., 2020; Kuai et al., 2021; Vannoy et al., 2021; Zheng et al., 2021), and the unique properties of the reaction environment (Li et al., 2014; Lee et al., 2019a, 2019b; Gao et al., 2019; Huang et al., 2021; Wang et al., 2021; Gong et al., 2022; Qiu et al., 2022). These features give rise to numerous applications (Wei et al., 2017; Yan et al., 2018; Zhu et al., 2019; Nie et al., 2020; Li Z. et al., 2021; Gunawardena et al., 2021; Yan, 2021; Zhang, 2021). Reaction acceleration is firmly attributed to the reactions at the solution/air interface (Yan et al., 2016; Lee et al., 2019b; Wei et al., 2020). Partial solvation of the reactants at the droplet surface has been proposed to increase the reaction rate constant at the interface compared to the bulk phase (Yan et al., 2016; Wei et al., 2020), a suggestion supported by multiple experimental results (Bain et al., 2015; Yan et al., 2016; Wei et al., 2020; Li et al., 2021b; Qiu et al., 2021) and limited high-level quantum mechanical modeling (Narendra et al., 2020). A detailed study of reaction rate constants in microdroplets showed that the acceleration factor (i.e., ratio of rate constant in confined volume vs. rate constant in bulk) is between 10^4^ to 10^6^ for all the bimolecular reactions investigated (Qiu et al., 2021). The partial solvation hypothesis explains reaction acceleration on the following basis: 1) the solvation energy of reactants is greater than that of the (more charge-dispersed) transition state, and 2) partial solvation (at the air/droplet interface) reduces solvation energies relative to complete solvation (in bulk). These solvation energy effects decrease the activation energy for interfacial reactions relative to bulk so producing accelerated reactions. Note that the first assumption holds for bimolecular reactions but not for unimolecular transformations, a fact consistent with the experimental observation that only bimolecular reactions show large rate acceleration effects (Qiu et al., 2021). One factor that can affect reaction rates without changing the rate constant is solvent evaporation, which increases concentrations (Rovelli et al., 2020). Other interfacial effects have been considered in explaining observed reaction acceleration (Fallah-Araghi et al., 2014; Yan et al., 2016; Lee et al., 2017, 2019a, 2019b, 2020; Zhou et al., 2018; Wei et al., 2020; Xiong et al., 2020; Huang et al., 2021), including the extreme pH at the droplet surface (Basuri et al., 2020; Huang et al., 2021), preferential orientation of reactants (Zhou et al., 2018; Narendra et al., 2020), and strong electric fields (on the order of MV/cm) at or near the microdroplet interface (Lee et al., 2019b, 2020; Xiong et al., 2020). The existence of the strong fields is supported by spectroscopic (Xiong et al., 2020) and computational (Leung, 2010; Kathmann et al., 2011; Yesibolati et al., 2020; Hao et al., 2022) data for aqueous microdroplets. The electric field lowers the energy barrier for reaction by stabilizing the transition state or by activating the reactant. Both effects accelerate reactions (Kreuzer, 2004; Aragonès et al., 2016; Fried and Boxer, 2017; Welborn and Head-Gordon, 2019; Ashton et al., 2020; Shaik et al., 2020). Moreover, reactive species can reasonably be proposed to be generated by strong electric fields causing unique reactions (Lee et al., 2019a, 2019b, 2020; Gao et al., 2019; Gong et al., 2022; Qiu et al., 2022).

Spontaneous oxidation and reduction without the addition of reducing/oxidizing agents has been reported in microdroplets (Lee et al., 2019a; Gao et al., 2019; Gong et al., 2022; Qiu et al., 2022), although the species responsible for these redox reactions is still under debate. Data from Zare and coworkers led them to conclude that the reactive species are the hydroxyl radical and hydrogen peroxide (Gao et al., 2019; Lee et al., 2019b, 2019a). These authors hypothesize that the high electric field at aqueous microdroplet interfaces can oxidize hydroxide to hydroxyl radicals, which can in turn generate hydrogen peroxide. They proposed that these reactive species can induce both reduction, e.g., acetophenone to 1-phenylethanol (Lee et al., 2019a), and oxidation, e.g., aromatic aldehydes to phenols (Gao et al., 2019). On the other hand, Zhang and collaborators postulated that electrons at the microdroplet air-water interface account for the reduction of doubly-charged ethyl viologen to the singly-charged radical cation and subsequent degradation reactions (Gong et al., 2022). Recently, our group suggested that water radical cations can be generated in organic-solvent microdroplets containing trace amounts of water and that they play a key role in the oxidation of aromatic sulfones to sulfonic acids in microdroplets (Qiu et al., 2022). Water radical cations are reported to be naturally abundant in pure bulk water (Ben-Amotz, 2011; Lee and Rick, 2011) and are generated by electric discharge in water vapor under otherwise mild conditions (Wang et al., 2021). Notably, in a study preceding this one, water radical cations produced in charged microdroplets were shown to trigger spontaneous oxidation (Qiu et al., 2022). The argument that water radical cation is a key reactive species is supported by the fact that the water radical cation has a higher oxidative power than either the hydroxyl radical or hydrogen peroxide in aqueous solution (Ma et al., 2014, 2018). The generation of water radical cations could occur by 1) the ionization of water due to the strong electric field at the microdroplet interface or 2) by hydrogen bond-induced electron transfer to form a water radical cation/anion pair (H_2_O∙∙∙OH_2_ ⇌ H_2_O^**+·**^ + H_2_O^**−·**^) (Ben-Amotz, 2011; Lee and Rick, 2011). The fleeting existence but high concentration of the water radical cation allows it to form adducts ([M + H_2_O]^**+·**^) by a displacement reaction with the sulfones, while subsequent 1,2-aryl migration and C-O cleavage yields sulfonic acids (Qiu et al., 2022). Interestingly, not only is oxidation accelerated by the reactive water radical cations, but it shows an extraordinarily high regioselectivity (>100 fold), presumably due to amplification by reaction acceleration of differences in substituent migratory aptitudes. Furthermore, water radical cation oxidation of an aromatic ketone was also shown (Qiu et al., 2022). These observations motivated us to investigate the generality of water radical cation adduct formation and associated oxidation.

To study this spontaneous oxidation, we utilized both nano-electrospray ionization (nESI), a common technique utilized in microdroplet reaction acceleration studies (Bain et al., 2015; Yan et al., 2016; Sahota et al., 2019; Wei et al., 2020) and desorption electrospray ionization (DESI) to generate charged microdroplets. DESI is an ambient ionization technique (Takáts et al., 2004; Cooks et al., 2006) that has found a wide application (Ifa et al., 2008; Morelato et al., 2013; Ifa and Eberlin, 2016; Zheng and Chen, 2016; Pirro et al., 2017). The micron-sized secondary droplets of DESI (Costa and Graham Cooks, 2008), facilitate reaction acceleration and its applications to on-line derivatization and small-scale organic synthesis (Girod et al., 2011; Morato et al., 2021). The compatibility of DESI-MS with automated high-throughput screening enables chemical analysis at throughputs better than one sample per second using high-density arrays (50 nL samples, up to 6,144 samples per array) (Morato et al., 2021). Applications include screening of organic reactions (Wleklinski et al., 2018; Jaman et al., 2019, 2020; Loren et al., 2019; Biyani et al., 2020; Ewan et al., 2020; Sobreira et al., 2020; Li et al., 2021a; Morato et al., 2021), label-free enzymatic assays (Morato et al., 2020; Kulathunga et al., 2022), and the generation of spectral libraries (Le et al., 2021). Here, we take advantage of high-throughput DESI to investigate adduct formation by water radical cations in a large compound set. We also performed a detailed study, using a small set of monofunctional model compounds on the mechanism of adduct formation and the subsequent oxidation.

## 2 Results and Discussion

### 2.1 High-Throughput Systematic Exploration of Molecular Ionization by Water Radical Cations

Given that sulfones have been shown to be ionized as [M + H_2_O]^**+·**^ (Qiu et al., 2022), the question of how general is this behavior must arise. Automated DESI-MS analysis allows rapid (<1 s per sample) generation of spectral libraries, as recently demonstrated utilizing a set of 20,798 non-proprietary compounds (Le et al., 2021). That study focused on the fragmentation of protonated ([M+H]^+^) compounds, but here the same MS spectral library was utilized to evaluate the nature of the molecular ion. Protonation and alkali ion attachment are the expected ionization reactions but the presence of the [M+H_2_O]^+^˙ ion is a previously unrecognized complication in its use for molecular weight determination by MS. This information was obtained by retrieving the intensities of ions with *m/z* values corresponding to [M+18]^+^˙ in the large set of polyfunctional library compounds with vast structural diversity. Note that a small contribution by artifactual peaks at this *m/z* value are to be expected. A schematic of the automated DESI-MS experiment is shown in Supplementary Figure S1.

**FIGURE 1:**
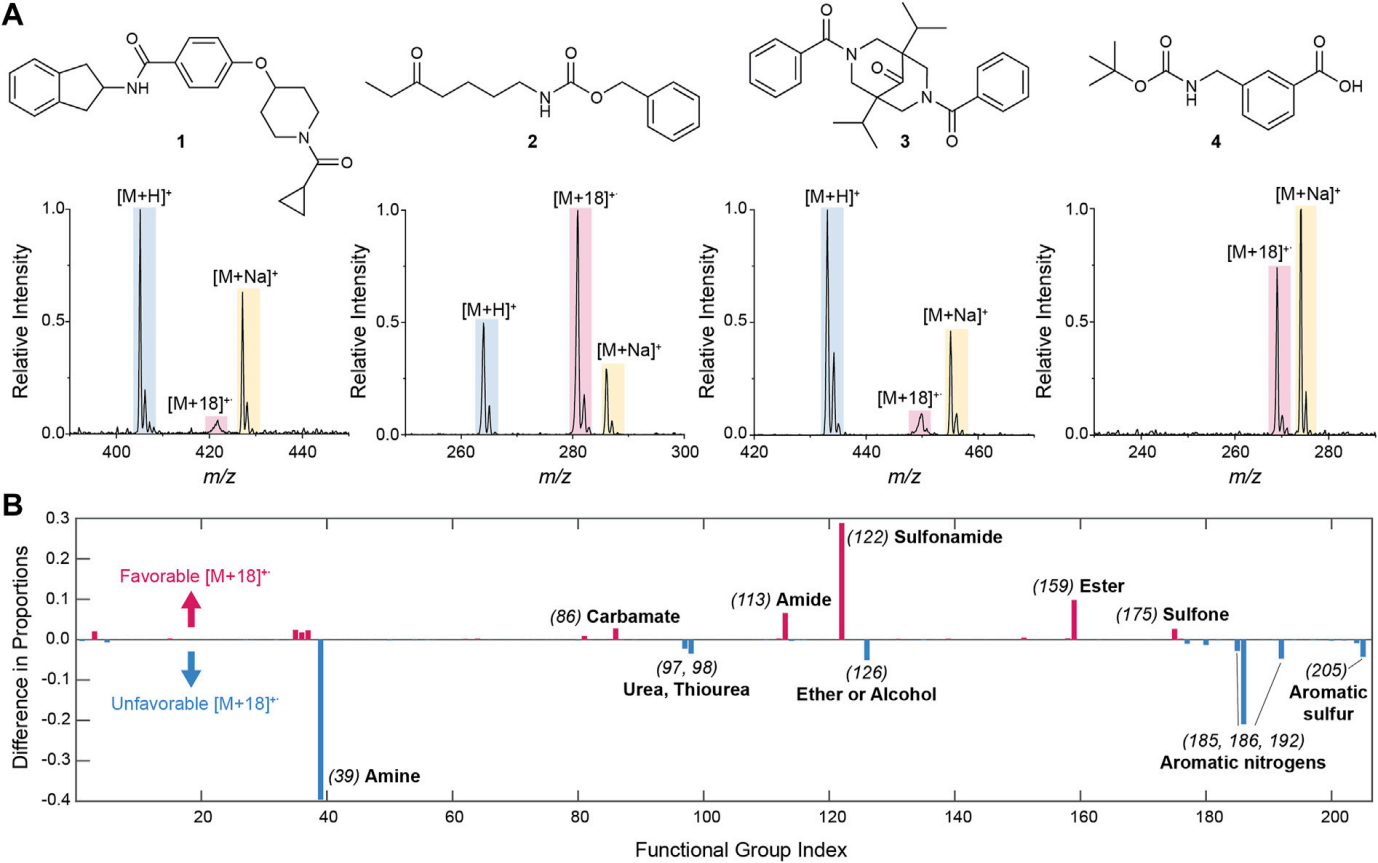
**(A)** Representative examples of mass spectra from the 20,798-compound library showing cases of high and low [M+18]^+^˙ intensity. Note the peak broadening in the low intensity cases. The structures of the molecules corresponding to the example spectra are also shown. **(B)** Effect of functional groups present in the molecule on the generation of [M+18]^+^˙ as estimated from the difference in the proportion of compounds with a functional group within the set of [M+18]^+^˙ hits and the same proportion in the complete library (Supplementary Equation S3). A difference of zero indicates no effect of the functional group. Significant positive differences indicate that the formation of the [M+18]^+^˙ species is favored by the presence of the functional group, whereas significant negative differences indicate a disfavoring effect. For the sake of simplicity, functional groups are indicated by indices specified in Supplementary Table S1.

To identify the presence of a peak, the intensity of the [M+18]^+^˙ species was compared to a blank and signal-to-noise ratio, SNR >3, was used to recognize the peak. Comparisons with the intensities of the [M+H]^+^, [M+Na]^+^, and [M+17]^+^ intensities were made as described in the Supplementary Material. We identified 3,683 hits with a substantial [M+18]^+^˙ peak compared to 16,662 and 14,121 compounds showing [M+H]^+^ and [M+Na]^+^, respectively. Some representative spectra (of compounds ***1***–***4***) corresponding to high and low [M+18]^+^˙ intensities are shown in Figure 1A. Note that the low intensity [M+18]^+^˙ peaks were in many cases broad, indicating that in those instances the interaction between the molecule and the water radical cation is weak, and the resultant ion is consequently fragile. Such a relationship between weakly-bound ion clusters and the peak broadening observed during mass analysis in quadrupole ion traps is well known (McClellan et al., 2002). Of course, the low intensity of the peak by itself is already an indication of the low stability of the [M+18]^+^˙ species. It is worth highlighting, however, that more than 65% of the hits identified showed intense (>30% relative intensity) [M+18]^+^˙ peaks, and that these all displayed unit resolution (characteristic resolution of the instrument utilized) indicating strong bonding between the molecule and the water radical cation, as previously demonstrated in the case of sulfones (Qiu et al., 2022).

To gain further insight into the molecular interactions leading to the generation of [M+18]^+^˙ species, we investigated the effect of the functional groups present in the molecules that gave hits. To achieve this we first extracted all the functional groups present in the complete library of compounds utilizing their SMILES strings as input and Ertl’s algorithm as previously described (Ertl, 2017; Le et al., 2021). Note that this algorithm designates functional groups as groups of special atoms (i.e., heteroatoms as well as *sp* ^2^, *sp*, and acetal carbons). Following this approach, a total of 205 functional groups were identified in the whole library (Supplementary Table S1). The proportion of any particular functional group within the library was then calculated by counting all compounds that have said functional group and dividing it by the total number of compounds in the library. In a similar manner, one can find the proportion of every functional group within the set of species that provided [M+18]^+^˙ hits (Supplementary Equations S1, S2). If there is no effect of a functional group in the generation of [M+18]^+^˙ ions, the proportion of compounds with that functional group in the overall library and in the [M+18]^+^ hits set is expected to be similar. By contrast, if those proportions differ significantly, this indicates that the presence or absence of the functional group influences the generation of a [M+18]^+^˙ species.

Clear trends were observed showing the influence of functional groups on the formation of water radical cation adducts (Figure 1B, Supplementary Table S2). In particular, the presence of sulfonamides, amides, esters and carbamates was found to significantly favor the generation of [M+18]^+^˙ species; this is in accordance with the expectation that H_2_O^**+·**^ will react with X=Y groups, and in agreement with the proposed mechanism of adduct formation (Wang et al., 2021; Qiu et al., 2022). The absence of basic nitrogen atoms in these functional groups reinforces this result. On the other hand, water radical cation adduct formation is relatively unfavorable for compounds containing basic nitrogen atoms (i.e., amines, ureas, and nitrogen-containing heterocycles). Both results indicate a competition between protonation and formation of the water radical cation adduct in which protonation is favored in the presence of basic groups and covalent adducts being formed with X=Y functionalities.

Note that the ionization of library compounds to give the radical cation form of the molecular ion (M^+^˙) was also investigated using similar searching and filtering strategies as for the [M+18]^+^˙ species. Overall, this radical cation was far less abundant than the water radical cation adducts. In fact, of all the compounds showing hits for the [M+18]^+^˙ species, only 132 (ca. 3% of the hits) showed a M^+^˙ peak, indicating that the observed adduct is likely a water radical cation derived species (M + H_2_O^+^˙), as proposed, and not the water adduct of the molecular radical cation (M^+^˙ + H_2_O).

### 2.2 Processes Leading to Water Radical Cation Adducts

**FIGURE 2:**
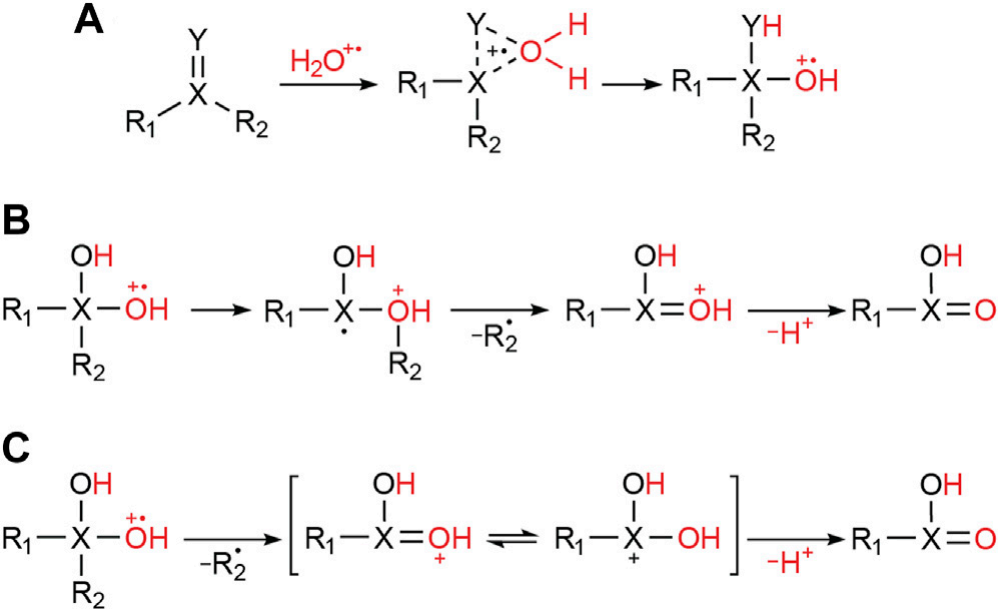
Possible processes for water radical cation adduct formation with a double bond-containing compound. **(A)** Direct attachment of the water radical cation (monomer or dimer) to the reactant. **(B)** Oxidation of the substrate by 1,2-migration and sigma bond cleavage. **(C)** Oxidation of the substrate by geminal diol cation formation after direct sigma bond cleavage.

It is known that the water radical cation forms as an O-O weakly bonded dimer (i.e., [H_2_O∙∙∙OH_2_]^+^˙) and that this species can react with various unsaturated compounds to form water radical cation adducts. Examples include alkenes (Zhang et al., 2020), benzene (Mi et al., 2022), acetone (Zhang X. et al., 2021), ethyl acetate (Wang et al., 2021) and various sulfones (Qiu et al., 2022). The variety of substrates that form adducts with the water radical cation and the spontaneous oxidation driven as a result of adduct formation, triggered our interest in systematically exploring the properties of water radical cation adducts generated in microdroplets. As mentioned before, these ions occur in the positive ion mass spectra as [M+18]^+^˙ species. Note that MS/MS data (Section 2.5 below) show the adduct to be strongly bound; several structures can be attributed to the general adducts, including a three-membered ring and a geminal diol structure (when Y is oxygen) (Figure 2A). The adduct can undergo 1,2-migration and radical induced bond cleavage (Figure 2B) or direct bond cleavage (when the bond is fragile, so generating a geminal diol cation, Figure 2C).

### 2.3 Mechanistic Study of Water Radical Cation Adduct Formation

**FIGURE 3:**
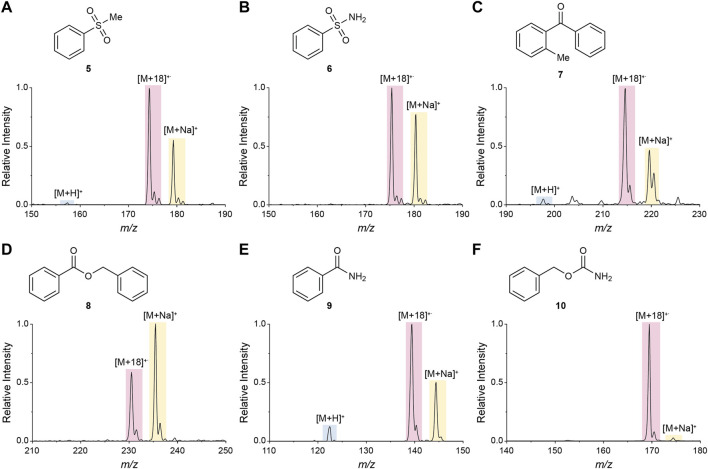
Mass spectra of six monofunctional compounds in nESI-generated microdroplets. Sulfone **(A)**, sulfonamide **(B)**, ketone **(C)**, ester **(D)**, amide **(E)**, and carbamate **(F)** functionalities were explored.

To further explore the effect of functional groups on the competitive generation of water radical cation adducts ([M+18]^+^˙) and protonated molecules ([M+H]^+^), we chose six monofunctional compounds (***5***–***10***) bearing different functional groups that were found to favor the generation of water radical cation adducts during the large compound library analysis (sulfone, sulfonamide, ketone, ester, amide, and carbamate). Methanolic solutions of these compounds were analyzed using nESI-MS and DESI-MS (commercial methanol was used as solvent). As expected from the compound library analysis, [M+18]^+^˙ species were observed in all cases (Figure 3 and Supplementary Figure S3), albeit with higher intensities (i.e., [M+18]^+^˙/[M+H]^+^ ratios) in nESI than in DESI, probably due to the smaller sizes and higher effective concentrations of the nESI-generated droplets.

The DESI-MS analysis was repeated using methanol with 0.1% formic acid as DESI solvent (same conditions of analysis as the library compounds). Under these conditions, the [M+18]^+^˙ species were still observed (Supplementary Figure S4), however with lower intensities than in the absence of formic acid. This indicates that the formation of [M+18]^+^˙ is unfavorable in microdroplets containing this acid and is consistent with the decrease in product conversion observed for the benzenesulfonamide oxidation after formic acid addition (Section 2.4 below), as is expected according to the proposed reaction mechanism. This effect likely comes from the properties of formic acid as a reducing agent (Garron and Epron, 2005; Choi et al., 2013; Al-Naji et al., 2020), allowing it to react directly with the oxidizing water radical cation, or to react as a proton donor to enhance the competitive direct protonation of the substrate (vs. intermediate adduct formation). Note that this effect of formic acid suggests that the number of compounds shown to be capable of generating substantial water radical cation adducts in the library analyzed is likely an underestimation.

**FIGURE 4:**
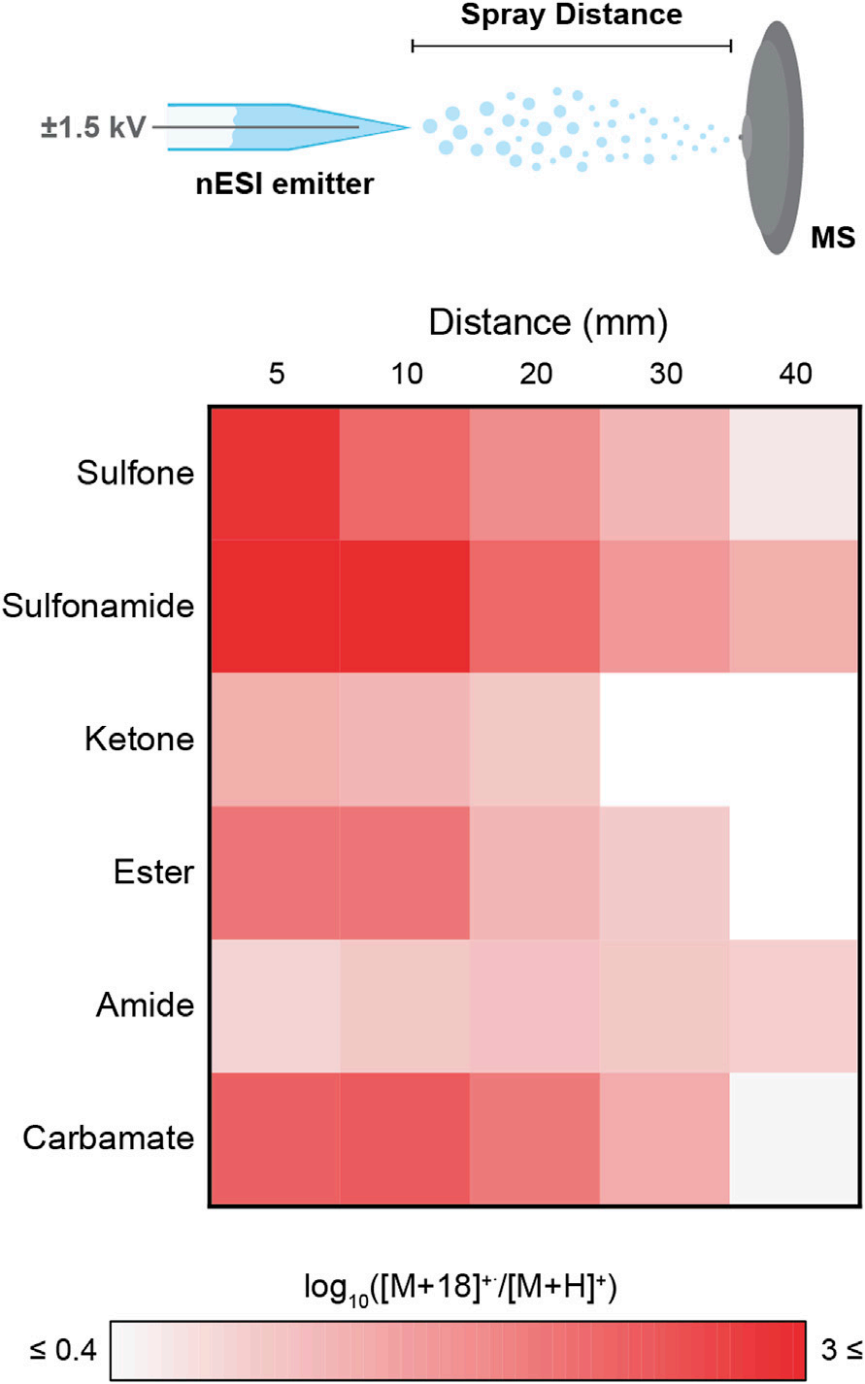
Effect of the functional group and spray distance (i.e., distance between the nESI emitter and the mass spectrometer inlet) on the generation of [M+18]^+^˙, monitored as the ratio [M+18]^+^˙/[M+H]^+^. Note the color scale is logarithmic to facilitate visual comparison. Results are for six representative monofunctional compounds identified by their functional groups: sulfone (benzyl methyl sulfone, ***5***), sulfonamide (benzenesulfonamide, ***6***), ketone (2-methylbenzophenone, ***7***), ester (benzyl benzoate, ***8***), amide (benzamide, ***9***), and carbamate (benzyl carbamate, ***10***).

The spray distance (i.e., microdroplet flight distance from the nESI emitter to the mass spectrometer inlet) was varied from 5 to 40 mm, while monitoring the ratio of [M+18]^+^˙/[M+H]^+^ as a proxy for the stability of the [M+18]^+^˙ species in the microdroplets (Figure 4). The strongest tendency to form [M+18]^+^˙ over [M+H]^+^ was found in the sulfonamide case which provided [M+18]^+^˙/[M+H]^+^ ratios as high as 10^3^. The sulfone, ester and carbamate also showed high ratios. All these results are in complete agreement with those obtained from the high throughput library exploration. In contrast, the model amide analyzed exhibited a low [M+18]^+^˙/[M+H]^+^ ratio (ca. 10), despite the favorability estimated with the compound library analysis. This minor discrepancy is likely the result of an increased basicity of the -CONH_2_ present in the model compound when compared to the -CO-NH-R (R ≠ H) moiety, which is the common amide structure within the library. Across all functional groups, increasing the spray distance (i.e., decreasing droplet size and increasing surface-to-volume ratios), resulted in lower [M+18]^+^˙/[M+H]^+^ ratios. This result contrasts with the negative ion data shown in Figure 6E below (Section 2.4). Several factors are at play here: 1) fragmentation of [M+18]^+^˙ to [M+H]^+^ by loss of a hydroxyl radical increases with distance and inverts the expected ratio, 2) conversion of [M+18]^+^˙ to oxidation products, and 3) competitive direct protonation of the molecule (M) during flight to provide [M+H]^+^.

### 2.4 Reactions Driven by Water Radical Cation Adducts

**FIGURE 5:**
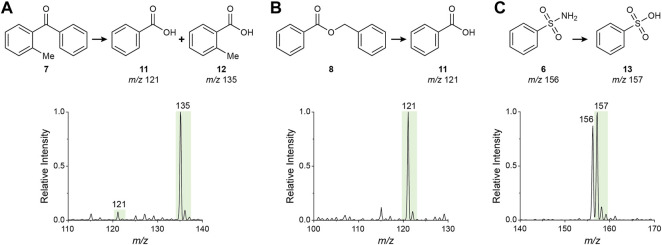
Negative ion mode nESI**-**MS spectra of methanolic solutions of **(A)** 2-methylbenzophenone **(*7*)**, **(B)** benzyl benzoate **(*8*)** and **(C)** benzenesulfonamide **(*6*)** showing the product(s) of oxidation (for the ketone and ester) and/or hydrolysis (for the ester and sulfonamide) driven by the water radical cation in microdroplets.

To investigate the oxidation reactions driven by the water radical cation adduct, we selected three of the six monofunctional model compounds ***6***–***8*** containing X=Y bonds namely 2-methylbenzophenone (***7***, ketone), benzyl benzoate (***8***, ester) and benzenesulfonamide (***6***, sulfonamide). Using nESI-MS, methanolic solutions of these compounds (20 mM) were analyzed in the negative ion mode. In all cases the corresponding products (***11***–***13***), which are benzoic or sulfonic acids, were detected (Figure 5) and their structures were confirmed by MS/MS (Supplementary Figure S5). Formation of these products is proposed to follow the processes shown in Figure 2 and the specific mechanism for each case is illustrated in Supplementary Figure S6. Note the transition from the positive to the negative ion mode. Also note the strong product preference in the ketone oxidation (i.e., benzoic acid vs. 2-methylbenozic acid; Figure 5A) is presumably derived from the steric hindrance of the methyl group at the *ortho* position which modifies the normal almost equal phenyl/tolyl migratory aptitude (Supplementary Figure S7) (Aureliano Antunes et al., 2005). This effect is further amplified by the reaction acceleration phenomenon in microdroplets, thus providing a remarkably high 10-fold regioselectivity.

**FIGURE 6:**
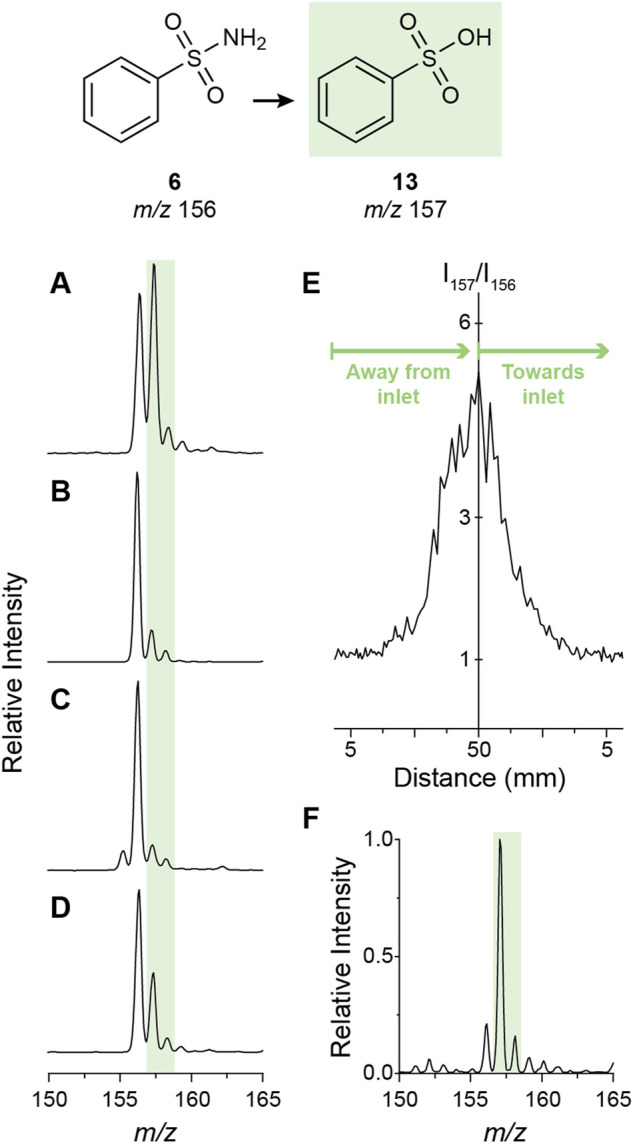
Mechanistic study of the microdroplet oxidation of sulfonamide ***6***. **(A)** through **(D)** show the effect of solvent composition on the amount of oxidation product observed using nESI-MS in the negative ion mode. Solvents explored were methanol **(A)**, methanol dried over MgSO_4_ **(B)**, 1:1 methanol-water **(C)**, and methanol with 0.1% formic acid **(D)**. The effect of the spray distance (i.e. distance from the nESI emitter to the mass spectrometer inlet) is shown in **(E)**. Note that from left to right the graph indicates moving away from the inlet (5–50 mm) and then back (50–5 mm). The mass spectrum corresponding to the maximum spray distance of 50 mm shows maximum conversion **(F)**.

The simultaneous detection of the sulfonamide (reactant) and the corresponding sulfonic acid (product) in the negative ion mode spectrum (Figure 5C) allows exploration of this transformation by monitoring the reaction conversion under different conditions. One factor explored was the solvent composition. Compared to commercial methanol, the addition of a desiccant (MgSO_4_) completely eliminated oxidation (Figure 6B), demonstrating the importance of water in the reaction. However, the addition of water (50% v/v) also shut off the reaction (Figure 6C), possibly by converting the water radical cation to the hydroxyl radical (Ma et al., 2014, 2018). Thus, trace amounts of water enhance the water radical cation mediated oxidation reaction. This outcome supports the suggestion that the sulfonamide hydrolysis is driven by the water radical cation rather than being due to direct hydrolysis by hydroxide. This conclusion is also supported by the experimental results that 1) the water dimer radical cation is observed in the spray of sulfonamide solution (Supplementary Figure S2) and 2) introduction of water dimer radical cation into the sulfonamide solution boosted the transformation to sulfonic acid (Supplementary Figure S8). It was observed also that adding formic acid traces (0.1%) to the methanolic solution reduced the reaction conversion (Figure 6D), in direct agreement with the effect of this acid in the abundance of the water radical cation adduct as previously discussed. The effect of the spray distance was also evaluated. Over the range explored (5–50 mm) larger distances provided higher conversion (Figure 6E; cf. Figure 6F at 50 mm and Figure 6A at 5 mm) with the maximum distance leaving virtually no unconverted reactant. This phenomenon has been reported for several microdroplet reactions and has been linked to the smaller sizes (and consequently larger surface-to-volume ratios) of the droplets with increased evaporation at longer flight times (Bain et al., 2015; Marsh et al., 2019).

The observed sulfonic acid product was characterized online *via* MS/MS (Supplementary Figure S5), and off-line by IR after microdroplet collection. To obtain a substantial amount of product, a multiplexed nESI sprayer containing 16 nESI emitters was built (Supplementary Figure S9; Supplementary Material for experimental details). From 2 mg of starting sulfonamide material, 1.7 mg of solid was collected when spray distance was 30 mm to optimize both collection efficiency and yield. The IR spectrum of the collected sample showed the characteristic O-H stretch of benzenesulfonic acid at 2,700–3,000 cm^−1^, a feature which is absent from the spectrum of the starting material (Supplementary Figure S10). The product yield in the collected material was estimated as 6.5% by MS analysis after correction for the ionization efficiency differences between reactant and product (Supplementary Figure S11; Supplementary Material for details). Note that microdroplet reaction yields can be increased using a solvent recycling system (Nie et al., 2020).

### 2.5 MS/MS Characterization of Water Radical Cation Adducts

The structures of the [M+18]^+^˙ species were probed using tandem mass spectrometry (MS/MS). Several collision energies as well as three different DESI solvents (methanol with 0.1% formic acid, methanol, and methanol-*d*_4_), were utilized. The major product ions and corresponding neutral losses are summarized in Table 1 (for spectra Supplementary Figure S12). Overall, the neutral loss of hydroxyl radical (−17) in MS^2^ is a general fragmentation channel of the [M+H_2_O]^+^˙ species, indicating again that these ions are not weakly-bound water adducts of radical cations. This is also reinforced by the fact that in many cases the adducts fragment to other product ions generated by cleavage of covalent bonds within the original compound structures (e.g., benzyl carbamate water radical cation fragments yielding a tropylium ion at *m/z* 91). Furthermore, upon ramping the collision energy (0–30 arbitrary units), it was observed that the six adducts formed are not weakly bound (as it would be in the case of the water adduct of a molecular radical cation) because they do not show significant fragmentation until high energies (≥20), and even then, a significant precursor ion population remains unfragmented (Supplementary Figure S13). Using methanol-*d*_4_, several adduct species were obtained, in particular [M+H_2_O]^+^˙, [M+HDO]^+^˙, and [M+D_2_O]^+^˙, detected at *m/z* values of [M+18]^+^˙, [M+19]^+^˙ and [M+20]^+^˙, respectively (Supplementary Figure S15). Analogously to the cases using non-deuterated solvent, the main fragmentation pathway of the [M+D_2_O]^+^˙ ions was the loss of DO˙, while neutral loss of HDO and D_2_O was also observed, especially in the sulfone and sulfonamide (Table 1). The presence of these multiple fragments agrees with the proposed existence of multiple water radical cation adduct structures (Figure 2).

**TABLE 1:** Main product ions and neutral losses (in parenthesis) of the [M+18]^+^˙ ions as determined by DESI-MS/MS analysis.*

Compound	MeOH +0.1% FA precursor ion: [M+18]^+^˙	MeOH precursor ion: [M+18]^+^˙	MeOH-*d*_4_ precursor ion: [M+20]^+^˙
Sulfone	157 (−17), 156 (−18), 142 (−32), 101 (−73)*, 96 (−78)	157 (−17), 156 (−18), 142 (−32), 101 (−73)*, 96 (−78)	158 (−18), 157 (−19)*, 156 (−20)
Sulfonamide	158 (−17), 157 (−18), 143 (−32)*	158 (−17), 157 (−18), 143 (−32)*	159 (−18), 158 (−19), 157 (−20)*, 142 (−35)
Ketone	197 (−17)*, 196 (−18), 182 (−32), 179 (−35), 161(−53), 158 (−56), 136 (−78)	197 (−17)*, 196 (−18), 182 (−32), 179 (−35), 161(−53), 158 (−56), 136 (−78), 100 (−114)	198 (−18)*
Ester	213 (−17), 212 (−18), 194 (−36), 186 (−44)*, 184 (−46)	213 (−17)*, 212 (−18)	214 (−18)*, 213 (−19), 212 (−20)
Amide	122 (−18)*	122 (−18)*	123 (−18)*, 122 (−19), 121 (−20)
Carbamate	152 (−17)*, 91 (−78)	152 (−18)*, 91 (−78)	153 (−18)*, 152 (−20), 151 (−20), 89 (−82)

*Three solvent systems were explored, methanol (MeOH) with 0.1% formic acid (FA), pure MeOH, and deuterated methanol (MeOH-*d*_4_). For the first two cases the [M+18]^+^˙ ion was selected as precursor ion, whereas for the last one, the [M+20]^+^˙ species was fragmented. The compounds utilized are identified by their functional groups: sulfone (benzyl methyl sulfone, ***5***), sulfonamide (benzenesulfonamide, ***6***), ketone (2-methylbenzophenone, ***7***), ester (benzyl benzoate, ***8***), amide (benzamide, ***9***), and carbamate (benzyl carbamate, ***10***). The most intense fragment in each MS/MS, spectrum is indicated by *.

### 2.6 Isomeric Forms of Water Radical Cation Adducts

Interestingly, during the MS/MS analysis of one of the model compounds, benzoyl benzoate (***8***), significant differences were observed between the spectra obtained when using methanol with 0.1% formic acid and neat methanol (Table 1), with more product ions being observed in the presence of the acid (note that the new fragments were absent from the corresponding experimental blanks under identical conditions). These differences held true over a large range of collision energies, up to the energy where no precursor ion population remained, eliminating any effect of energy differences as an explanation of this phenomenon. These observations strongly suggest that the addition of the acid is inducing the formation of an isomeric [M+18]^+^˙ species (Supplementary Figure S15). A potential isomer would result from protonation of the compound, favored by formic acid, followed by hydroxy radical addition. Such a process agrees with reported redox phenomena involving the generation of hydroxyl radicals at the microdroplet surface thanks to the action of high energy fields on hydroxide ions (Gao et al., 2019; Lee et al., 2019b, 2019a, 2020). The direct conversion of H_2_O^+^˙ to hydroxyl radicals may contribute, too. The formation of [M+18]^+^˙ species has been proposed to be favored by the promiscuity of electrons at the air-water interface (Ben-Amotz, 2011) and it correlates with molecular dynamics results suggesting an enhanced surface concentration of hydroxyl radicals compared to the one in aqueous bulk phase (Roeselová et al., 2004). Thus, the presence of this particular isomeric [M+18]^+^˙ ion might represent the coexistence of two mechanisms leading to accelerated redox chemistry in microdroplets. One, as described here, involves the water radical cation, a short-lived but abundant species spontaneously generated in water, and adduct formation with X=Y moieties as an intermediate step in an overall oxidation process; the second, involves hydroxyl radicals generated under highly energetic conditions at the droplet interface.

## 3 Conclusion

Water radical cation adduct formation was found to be a general phenomenon for X=Y functionalized molecules through the analysis of a large (>20,000) set of complex polyfunctional compounds, which represents 18% occurrence in all ca. 21,000 molecules and more than 65% in the set of X=Y compounds. Tandem mass spectrometry revealed that the adducts are covalently bound, and a closer examination of a small subset of model monofunctional molecules suggested that gem diol structures are involved. Upon exploration of a model ester (***8***), we identified structural differences in the generated [M+18]^+^˙ ion upon addition of formic acid traces to the microdroplet solvent. This observation suggests that there are two processes that compete in the generation of this particular [M+18]^+^˙ species, the major one being direct H_2_O^+^˙ addition, while the minor process involves OH˙ addition followed by protonation. In other words, there is evidence for two reactive oxidizing agents (H_2_O^+^˙ and OH˙). Note that OH˙ has been previously suggested to have this role (Gao et al., 2019; Lee et al., 2019b, 2019a) while H_2_O^+^˙ has only recently been identified as a strong oxidizing agent in microdroplets (Qiu et al., 2022). The competition between these two species is still to be fully explored. Similarly, some experimental factors, especially the emitter-inlet distance effect on the adduct generation, are incompletely elucidated.

The possibility of using this chemistry to generate small amounts of oxidized products without any external oxidants is clear. Note that these reactions occur under very mild conditions and are accelerated by such large factors that the corresponding reactions are not even observable in bulk solution. Extensive research has been devoted over the past decades to find green oxidation approaches for diverse functionalities (Goti and Cardona, 2008; Jiao and Stahl, 2019). In particular, the use of green solvent alternatives, the absence of catalysts, and reaction under mild conditions are relevant factors for efficient and environmentally-friendly oxidation (Yu et al., 2012; Amaniampong et al., 2017; Khan et al., 2022). All these requirements are fulfilled by the microdroplet oxidation process described in this work, which adds advantages such as the need for no external oxidizing agents, the wide applicability to various chemical functionalities, and the enhanced reaction rates as well as increased regioselectivities (Qiu et al., 2022). Furthermore, leveraging the properties of automated DESI-MS (Morato et al., 2021) or spray solvent recycling systems (Nie et al., 2020), the microdroplet-generated oxidation products could be easily screened and collected (nanogram scale) in a high-throughput fashion, or scaled up to obtain yields in milligram-gram amounts, respectively.

Similarly, the spontaneous oxidation induced by the water radical cation has several implications for atmospheric and aerosol chemistry, topics that involve sub-micrometer size droplets (e.g., spray and organic aerosols) (Gantt and Meskhidze, 2013; Ault and Axson, 2017; Kirpes et al., 2018). Although an enhanced rate of oxidation has been reported in aerosols *via* other mechanisms and other reactive species (e.g., oxygen, ozone, hydroxyl radicals or hydrogen peroxide) (Glasius et al., 2000; Richards-Henderson et al., 2016; Liu et al., 2020; Angle et al., 2021), we suggest that the possible role of the water radical cation chemistry is worthy of attention. The effects of this radical species might be significant considering the generality here demonstrated for X=Y containing compounds (common moieties in VOCs). Thus, the characterization and quantification of molecular composition in aerosols (Jeong et al., 2011) may be affected by the adduct formation with water radical cations and subsequent oxidation processes, especially in the case of organosulfate (Farmer et al., 2010; Tao et al., 2014; Wang et al., 2017) or carbonyl group-containing molecules (Kautzman et al., 2010; Takahama et al., 2013).

Finally, the facile water radical cation adduct formation seen in this study has direct implications for chemical analysis using mass spectrometry, particularly with regards to the automated chemical annotation of compounds. Currently, the identification of molecules (in the positive ion mode) is almost exclusively based in seeking the protonated form of the molecules with due consideration of sodium ([M+Na]^+^) and potassium adducts ([M+K]^+^) (Jaeger et al., 2017; Domingo-Almenara et al., 2018; Stricker et al., 2020). Adducts with water radical cation are not part of the identification of molecules in samples examined by MS. Remarkably, as shown in this work, these adducts represent significant peaks, in a substantial fraction of cases with greater intensities than the commonly searched protonated and sodiated forms of the molecules. Note that the leveraging of the spontaneous water radical cation adduct formation to obtain increased sensitivity for compounds with low ionization efficiencies, such as acetone (Zhang X. et al., 2021), may represent a novel derivatization-free strategy for mass spectrometry analysis.

## Data Availability

The original contributions presented in the study are included in the article/Supplementary Material, further inquiries can be directed to the corresponding author.
