# Supplementary material for: Spontaneous Water Radical Cation Oxidation at Double Bonds in Microdroplets
Source: Front Chem. 2022 Apr 26;10:903774. doi: 10.3389/fchem.2022.903774 (PMC9086510; doi:10.3389/fchem.2022.903774)
Supplement: Supplementary file 1 [file DataSheet1.docx]

Supplementary Material

Spontaneous Water Radical Cation Oxidation at Double Bonds in Microdroplets

Lingqi Qiu^†^, Nicolás M. Morato^†^, Kai-Hung Huang, and R. Graham Cooks*

Department of Chemistry and Center for Analytical Instrumentation Development, Purdue University, West Lafayette, IN 47907, USA

*** Correspondence:**R. Graham Cooks
[cooks@purdue.edu](mailto:cooks@purdue.edu)

^†^ These authors contributed equally to this work and share first authorship.

1. **Experimental Section**

## Chemicals

The six mono-functional compounds (viz. methyl phenyl sulfone, benzenesulfonamide, 2-methylbenzophenone, benzyl benzoate, benzamide, and benzyl carbamate were purchased from Sigma-Aldrich. Acetonitrile and methanol (LC-MS grade) were obtained from Fisher Scientific, while deuterated methanol (methanol-*d*_4_) was acquired from Cambridge Isotope Laboratories. All solvents were utilized as received unless otherwise stated. The non-proprietary compound library was a gift from Eli Lilly and Company.

## Automated high-throughput DESI-MS analysis

### High-throughput DESI-MS(/MS)

All samples were analyzed using the Purdue Make It system. The hardware, custom software and operation of this platform has been previously described (Sobreira et al., 2020; Morato et al., 2021). Briefly, solutions distributed in 384-well plates are spotted (50 nL per spot, 4 replicate spots per compound) onto PTFE-coated glass slides, which are then automatically analyzed using DESI-MS in a *spot-to-spot* fashion (ca. 500 ms spent on each spot) after the sample positions are calibrated using dye marks in the corners of the PTFE-coated slide. The final spectral result for each compound was obtained utilizing the average of four sample replicates. MS/MS experiments are performed in a similar manner, albeit with a lower throughput. In this case 10 s/sample was selected as MS/MS analysis time.

For the model compounds, 20 mM methanolic solutions were used as samples. Several solvents were explored, including methanol, methanol with 0.1% formic acid, and methanol-*d*_4_. A linear quadrupole ion trap (LTQ XL, Thermo Scientific) coupled to a 2D DESI stage (Prosolia) was utilized. The DESI spray voltage was set to 4.5 kV, the nitrogen gas pressure to 150 psi, the solvent flow rate to 2.75 µL/min, and the extended capillary temperature to 300 °C. MS/MS analyses were performed using collision induced dissociation (CID), with helium as the collision gas. An isolation window of *m/z* 1.5 units, an activation time of 30 ms, and a Mathieu *q* value of 0.25 were used. Several collision energies from 0 to 30 arbitrary normalized units were explored with all compounds.

### Data filtering

A signal-to-blank ratio (SNR) cut-off was implemented, setting the threshold to positively identify an [M+18]^+^˙ species at SNR ratios higher than 3. Then, filters were implemented related to the relative intensity of the [M+18]^+^˙ signal with respect to those for [M+H]^+^ and [M+Na]^+^. Both thresholds were set empirically at 10%, meaning that only compounds with both [M+18]^+^˙/[M+H]^+^ and [M+18]^+^˙/[M+Na]^+^ ratios above 0.1 would be considered acceptable. Note that when the protonated molecule or its corresponding sodium adduct were not observed, the corresponding filter was not implemented. Finally, [M+18]^+^˙ hits were filtered using the [M+17]^+^ intensity, to discard isotopic peaks of species identified at *m/z* values corresponding to [M+17]^+^, accepting only molecules with [M+18]^+^˙/[M+17]^+^ higher than 0.5. In all cases the extraction and processing of ion intensities for each spotted sample were carried out automatically using a combination of Python and MATLAB based scripts.

A similar strategy was implemented for identification of M^+^˙ species: SNR > 3, M^+^˙/[M+H]^+^ > 0.1, M^+^˙/[M+Na]^+^ > 0.1, and M^+^˙/[M-1]^+^. For identification of [M+H]^+^ and [M+Na]^+^ only the SNR filter was utilized.

### Equations for the calculation of functional group effects

For a given functional group (FG), the overall proportion of compounds with said FG within the library (OP_FG_) is calculated as:

${OP}_{FG}=\frac{Number of compounds with FG in the library}{Total compounds in the library}=\frac{Number of compounds with FG in the library}{20,798} \boldsymbol{(Equation S}\boldsymbol{1)}$

For a given FG, the proportion of compounds with said FG within the set of [M+18]^+^˙ hits (SP_FG_) is calculated as:

${SP}_{FG}=\frac{Number of compounds with FG in the hits set}{Number of compounds in the hits set}=\frac{Number of compounds with FG in the hits set}{3,683} \boldsymbol{(Equation S}\boldsymbol{2)}$

The effect of a particular FG on the generation of a [M+18]^+^˙ ion is estimated by comparing the proportion of compounds with the FG in the set of [M+18]^+^˙ hits and in the complete library (ΔP_FG_). A large positive difference indicates that the FG favors the formation of a [M+18]^+^˙ species, a large negative effect indicates that the FG hinders the formation of a [M+18]^+^˙ species, and no or little difference indicate that the FG has little to no effect on the generation of a [M+18]^+^˙ species.

$$\Delta P_{FG}={SP}_{FG}-{OP}_{FG} \boldsymbol{(Equation S}\boldsymbol{3)}$$

## Water radical cation adduction and oxidation study using nESI-MS

### Experimental details

All solutions used in the nESI microdroplet reactions were prepared in methanol at 20 mM concentrations, unless otherwise noted. The nESI emitters utilized had a 20 μm internal diameter and were made in house from borosilicate glass capillaries (1.5 mm O.D.; 0.86 mm I.D.; 10 cm length) using a micropipette tip puller from Sutter Instruments. The spray voltage was ± 1.5 kV with the spray distance as 5 mm unless otherwise stated. The operating parameters of the Thermo Scientific LTQ linear ion trap mass spectrometers were as follows: capillary temperature 150 °C; capillary voltage 15 V; tube lens 65 V.

### nESI droplets collection

To increase the scale of microdroplet reactions in nESI, a multiplex nESI set-up containing 16 identical nESI emitters (20 μm internal diameter) was built (**Figure S9A**). The center-to-center distance between adjacent sprayers was 8 mm. Stainless-steel electrodes (45 mm) were used, and their ends were soldered together in order to simultaneously apply the spray voltage.

For the droplet collection experiment, 20 μL of 20 mM (equivalent to 3.2 mg/mL) benzene-sulfonamide solution in methanol was loaded into each barrel. A voltage of – 1.5 kV, was applied to the electrodes and a grounded Indium Tin Oxide (ITO) plate was used as the substrate for the deposition (**Figure S9**). The spray distance was kept at about 30 mm to optimize collection efficiency and reaction conversion. After spraying for two hours, 20 μL of fresh solution was loaded to each barrel again and were sprayed for another two hours under the same conditions. The weight of the deposited solid (**Figure S9B**) was measured as 1.7 mg. The collected solid was dissolved in 2 mL of methanol (resulting concentration ca. 5 mM) for further characterization.

For Fourier transform infrared spectroscopy (FT-IR) analysis, 1 mL of the obtained solution was concentrated in vacuum and then analyzed using a Thermo Scientific Nicolet iS50 ATR spectrometer. The IR spectra of both the collected solid and the pure reactant are shown in **Figure S10**. The characteristic peaks of the sulfonic acid O-H stretch at 2700-3000 cm^-1^ were observed in the collected sample, indicating that the reaction occurred in the microdroplets.

To determine the amount of product in the collected sample, the ionization efficiency correction factor (*f*) was used to cancel the effects on the observed conversions due to the different ionization efficiencies of products and reactants. By definition, *f* is the ion intensity ratio of product to reactant divided by the actual concentration ratio of product to reactant:

$$f= \frac{I_{Product}/I_{Reactant}}{\left[ Product \right]/[Reactant]} \boldsymbol{(Equation S}\boldsymbol{4)}$$

The spike method (Nie et al., 2020; Qiu et al., 2021) was used to calculate *f* by measuring (i) the initial ion intensity ratio of product to reactant in the reaction mixture (S_1_), and (ii) the resulting ion intensity ratio after the addition of an equimolar amount of reagent with a volumetric ratio 1:1 (S_2_).

$$f=\frac{S_{1}S_{2}}{S_{1}-2S_{2}} \boldsymbol{(Equation S}\boldsymbol{5)}$$

To diminish the extent of microdroplet reaction during the analysis, the sample solution (5 mM) was diluted to 0.5 mM using methanol. A 0.5 mM benzenesulfonamide in methanol solution was prepared and used to prepare the spiked sample. The S_1_ ratio was calculated as 1.2 by averaging three separate measurements (RSD = 9%). The S­_2_ was calculated as 0.55 by averaging three independent replicates (RSD = 1%). Therefore, the corresponding *f* is 13.5 based on the equation S5, and the concentration ratio of the product to the reactant is 0.086. The weight of the product was 0.13 mg corresponding to 6.5% yield.

# Supplementary Figures and Tables

## Supplementary Figures

**
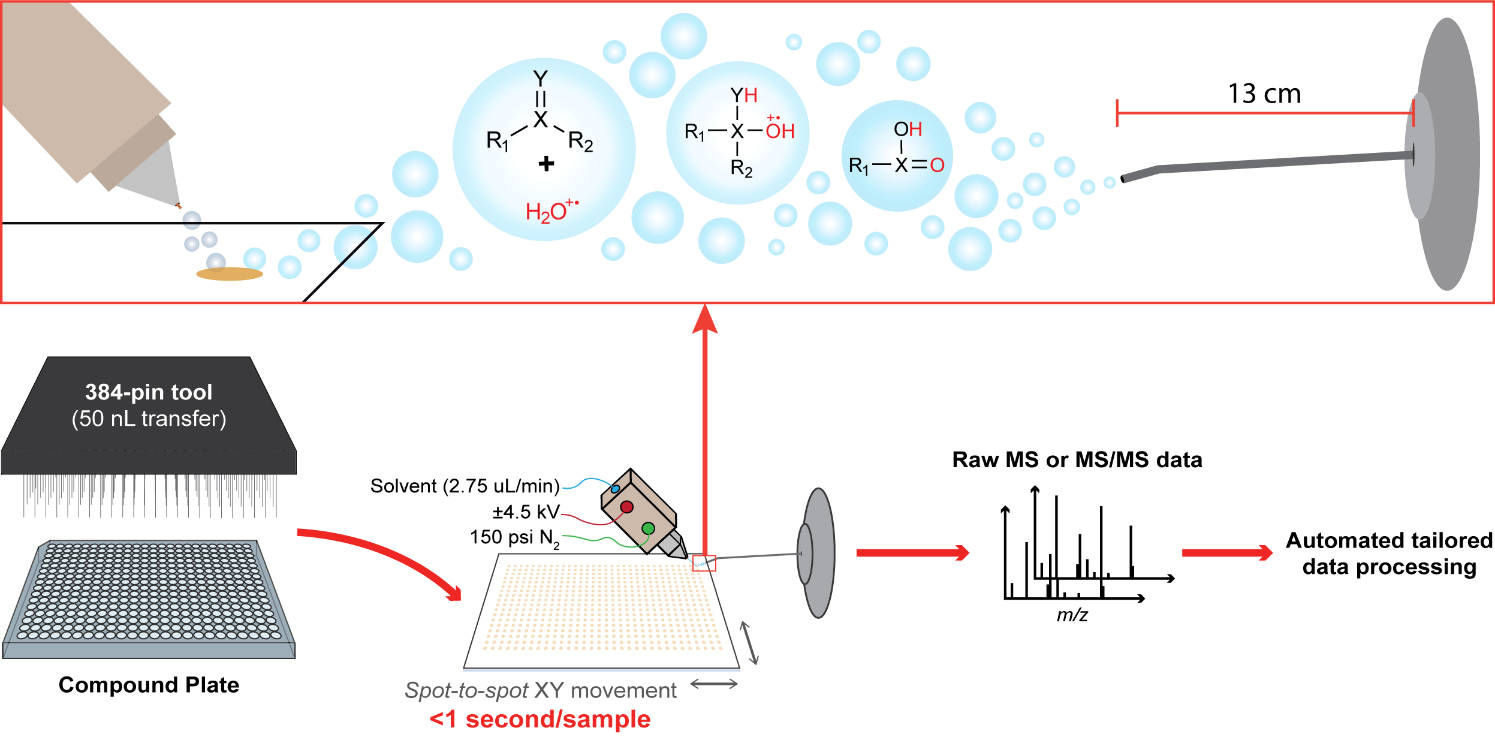
**

**Figure S1.** Schematic of the automated DESI-MS experiment. A 384-pin tool is used to transfer 50 nL of compound solutions from a 384-well plate to a PTFE-coated glass slide generating high-density arrays of up to 6,144 samples per plate. This plate is then automatically transferred and analyzed using DESI-MS at rates greater than 1 sample per second. The DESI spray generates secondary microdroplets (*see* zoomed-in inset) which carry the analyte (and in which reactions are accelerated *en route*) to the mass spectrometer. Shown here is the formation of the water radical cation adduct of an X=Y containing compound followed by its oxidation. The spectral data generated through the DESI-MS experiment is automatically processed to extract and analyze the information corresponding to *m/z* values of interest.


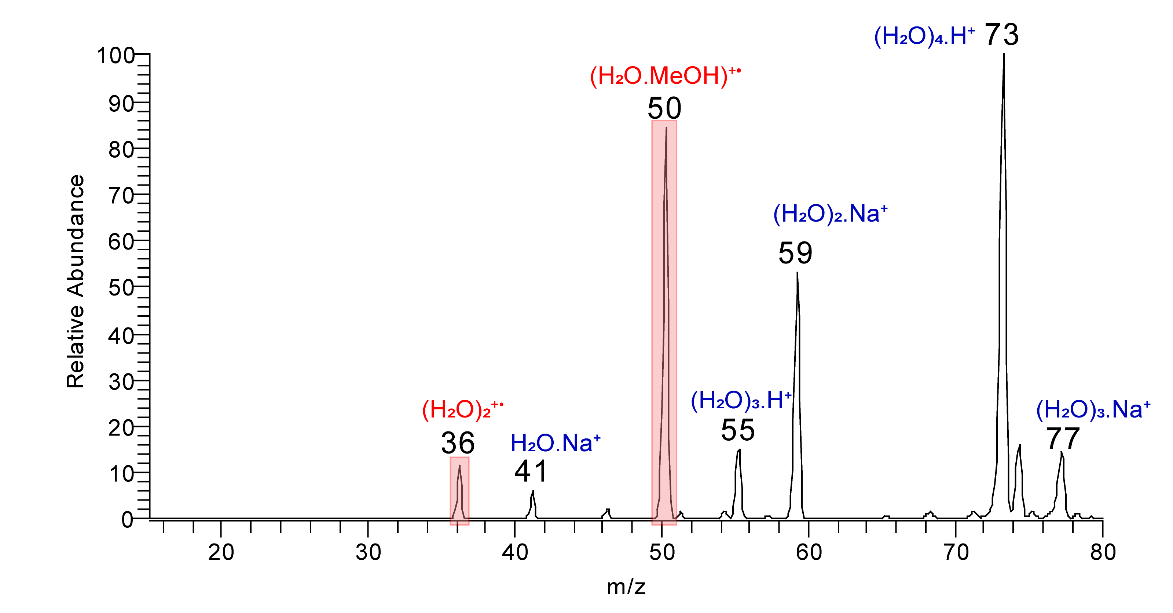


**Figure S2.** Low *m/z* range positive ion mass spectrum of a methanolic solution of benzenesulfonamide (20 mM). The species associated to the water radical cation are highlighted.

**
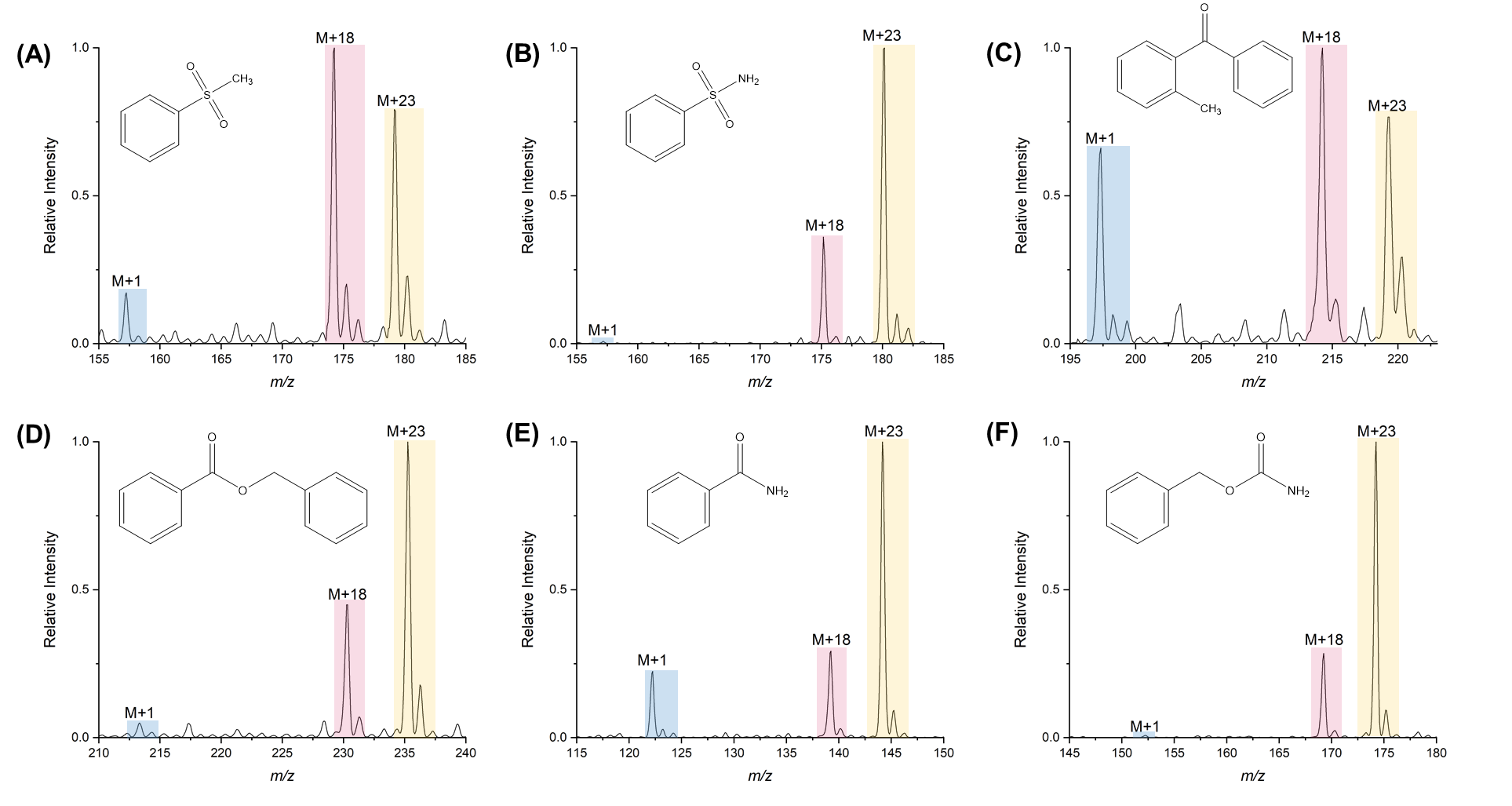
**

**Figure S3.** Positive ion mass spectra of six monofunctional compounds in DESI-generated microdroplets using methanol as DESI spray solvent. Sulfone (**A**), sulfonamide (**B**), ketone (**C**), ester (**D**), amide (**E**), and carbamate (**F**) functionalities were explored.

**
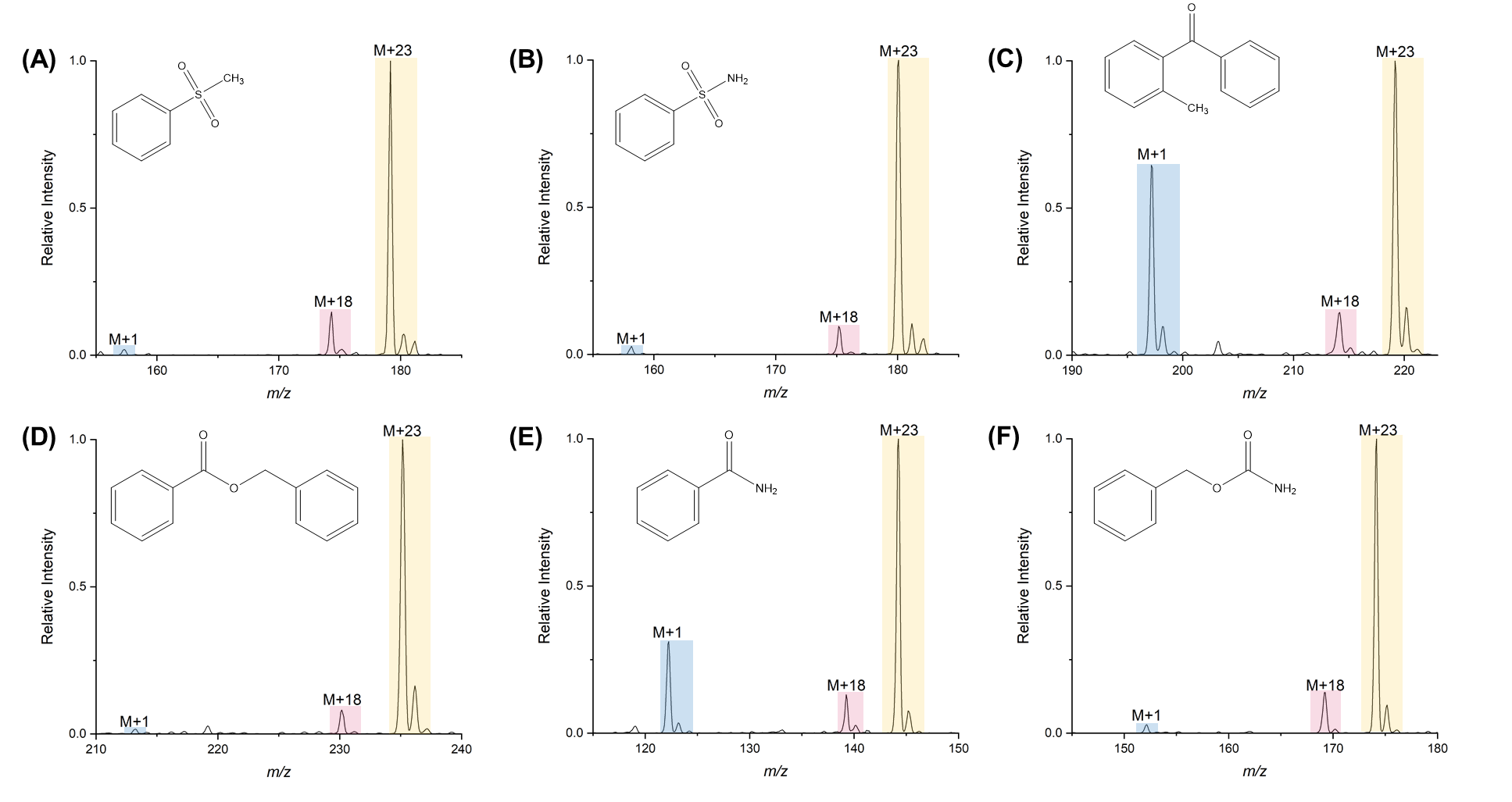
**

**Figure S4.** Positive ion mass spectra of six monofunctional compounds in DESI-generated microdroplets using methanol with 0.1% formic acid as DESI spray solvent. Sulfone (**A**), sulfonamide (**B**), ketone (**C**), ester (**D**), amide (**E**), and carbamate (**F**) functionalities were explored.


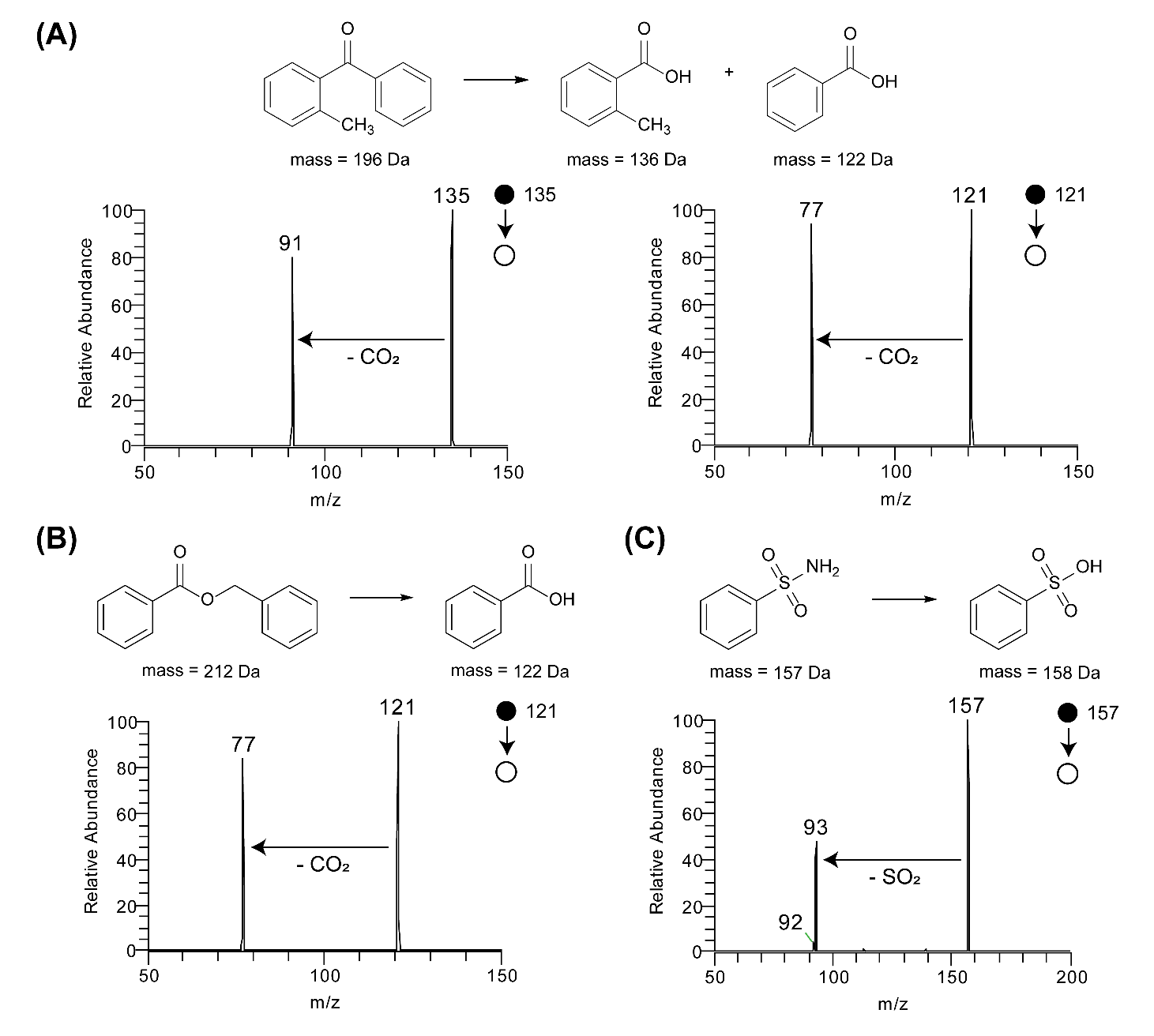


Figure S5. Tandem MS (MS^2^) negative ion mode analysis of the spontaneous oxidation products generated in microdroplets. In all cases the isolation width used was *m/z* 2.0. (A) MS^2^ spectra of ions at *m/z* 135 and *m/z* 121, showing characteristic CO_2_ loss (CID = 25). (B) MS^2^ spectrum of the ion at *m/z* 121, showing characteristic CO_2_ loss (CID = 25). (C) MS^2^ spectrum of the ion at *m/z* 157, showing characteristic SO_2_ loss. The observed small product ion at *m/z* 92 is produced by the fragmentation of traces of the deprotonated sulfonamide (*m/z* 156), which was incompletely isolated.

Figure S6. Proposed mechanism of the spontaneous oxidation in microdroplets via water radical cation adduct formation. (A) Reaction of the ketone by reacting with water radical cation, followed by 1,2-migration and C-O cleavage. (B) Reaction of the ester by reacting with water radical cation. Geminal diol cation is produced after a radical loss, and the final deprotonation generates the product. (D) Reaction of the sulfonamide by reacting with water radical cation. The geminal diol intermediate formation followed by the loss of ammonia gives the final product.

Figure S7. Explanation for the observed regioselectivity in the ketone oxidation. The migratory aptitude of the radical-based aryl migration depends on the transition state in the concerted mechanism (Aureliano Antunes et al., 2005). Two competing pathways for the ketone oxidation are depicted here, involving a 2-methylphenyl migration (pathway I) and phenyl migration (pathway II). The steric hindrance due to the *ortho-*methyl group in the transition state in the pathway increases the energy barrier, thus causing this pathway to be less favorable when compared to pathway I.


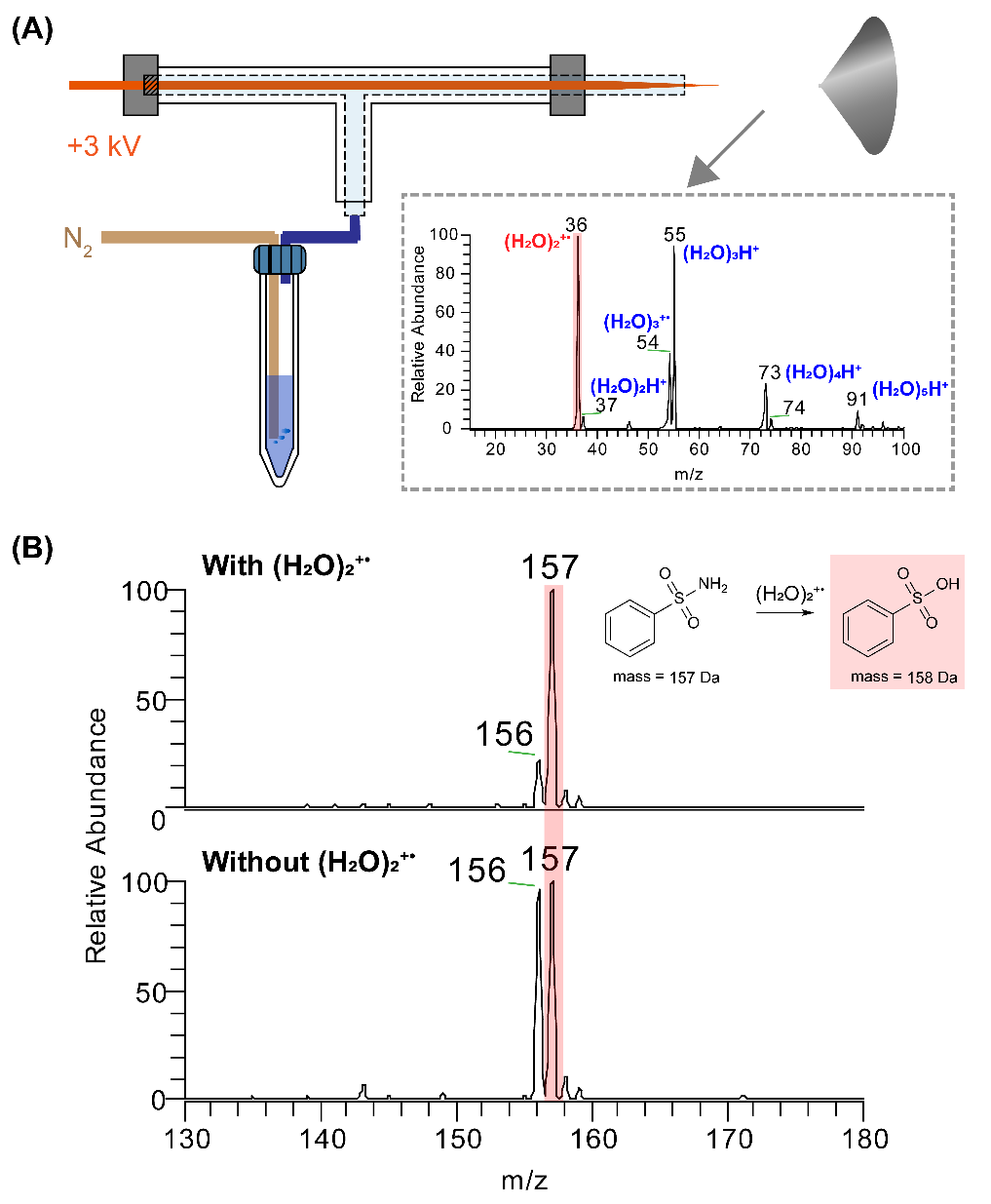


Figure S8. Introduction of water dimer radical cation to facilitate the conversion of sulfonamide to sulfonic acid. (A) Generation of water dimer radical cation by ionizing water vapor using a corona discharge apparatus (Qiu et al., 2022). A potential of +3 kV was applied to the electrode and the spray distance was kept at 25 mm. The generated water radical cations were detected in the positive mode. (B) The (H_2_O)_2_^+^˙ spray (spray voltage = 3 kV) was deposited onto the sulfonamide solution (10 μL, 20 mM) for 5 min, and the resulting residue was redissolved in 10 μL of MeOH for negative ion mode nESI-MS analysis. The same protocol was followed but using 0 kV spray voltage to provide a negative control. The data show clearly that the introduction of (H_2_O)_2_^+^˙ spray enhanced the reaction.


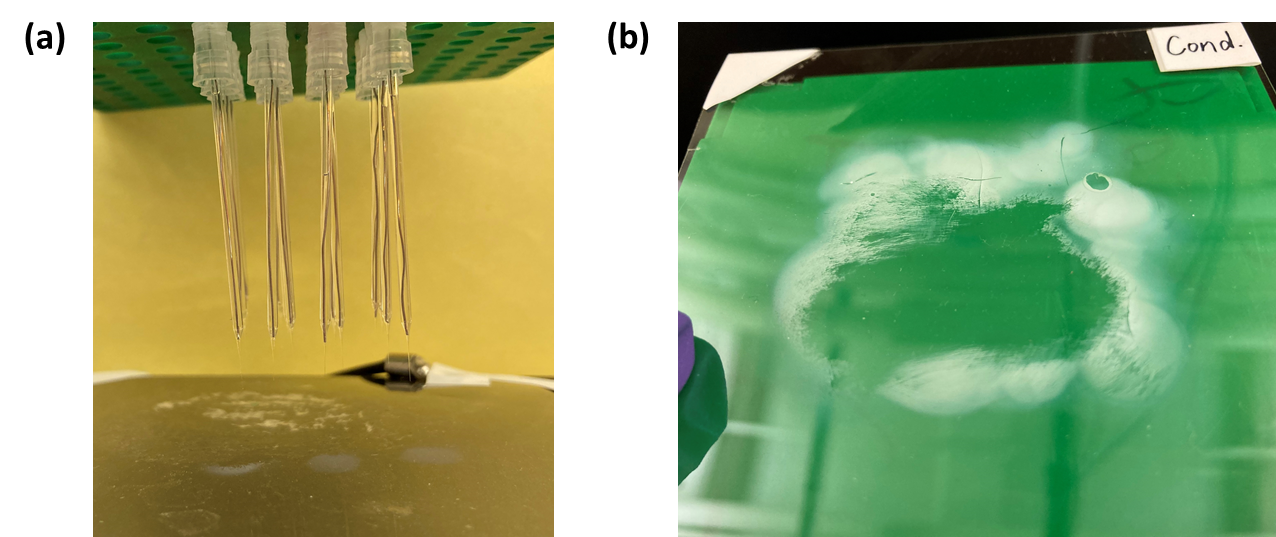


Figure S9. Photographs of (a) home-built multiplexed nESI set-up and (b) deposited reaction mixture. Note that for the photograph the spray distance in (a) was set at 15 mm, while during the experiment it was fixed at 30 mm.

**
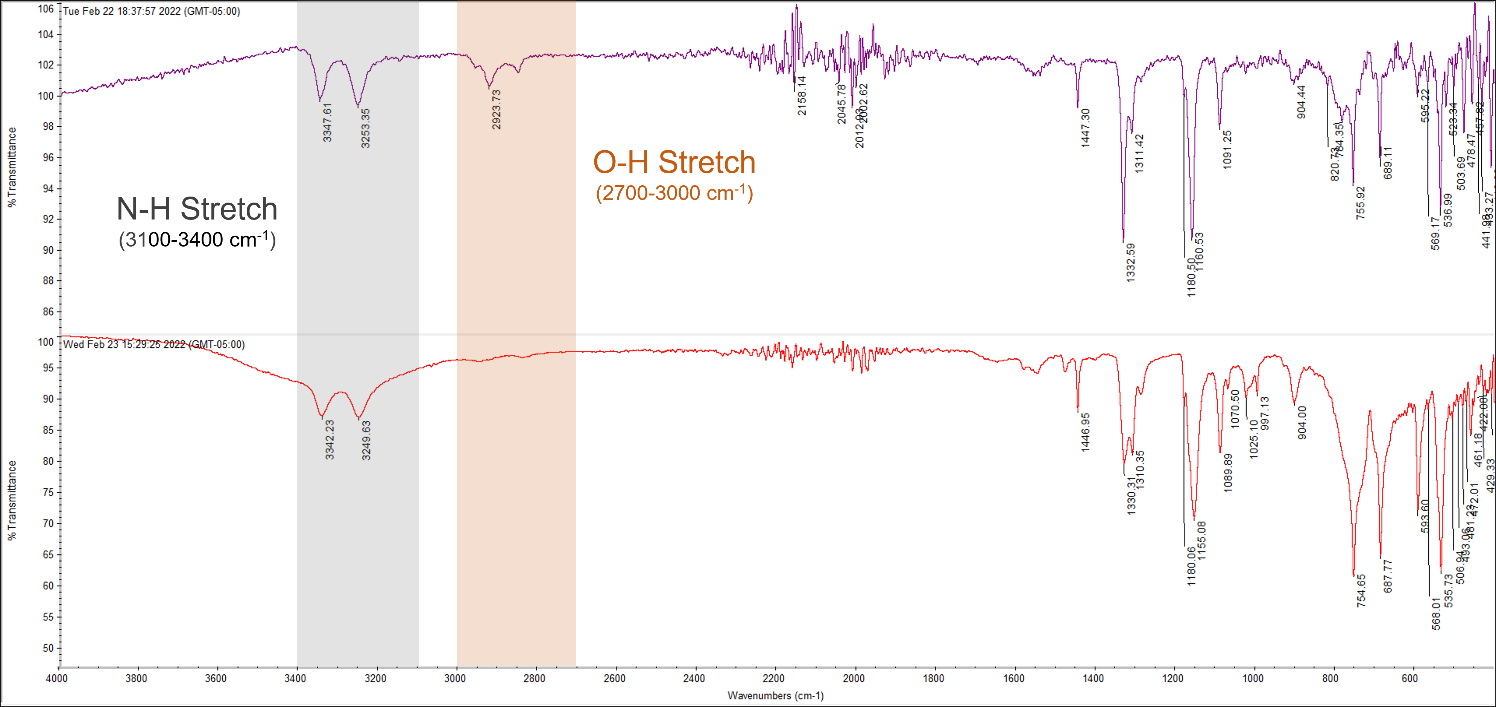
**

Figure S10. IR spectra of the collected solid (top channel, purple line) compared to pure reactant, benzenesulfonamide (bottom channel, red line). Note the characteristic O-H stretch signals of benzenesulfonic acid at 2700-3000 cm^-1^ in the top spectrum.


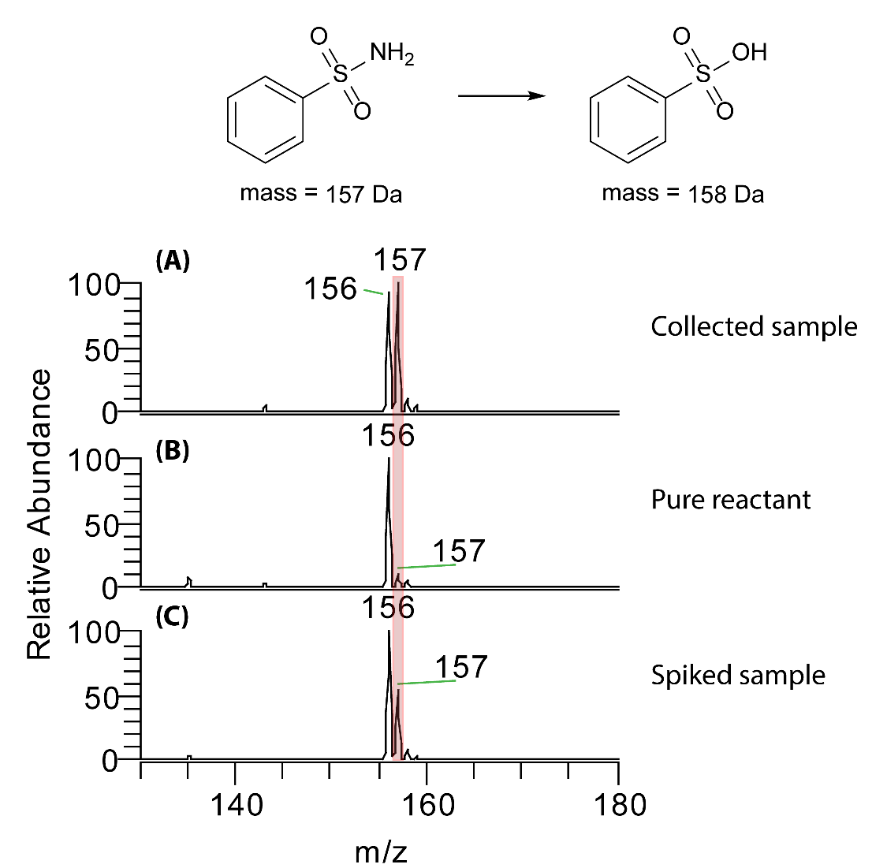


Figure S11. Negative ion mass spectra of (A) collected sample (diluted to 0.5 mM in methanol), (B) pure reactant (prepared as 0.5 mM in methanol), (C) the spiked sample, which was prepared by 1:1 mixing of the solution of the collected sample and pure reactant.

**
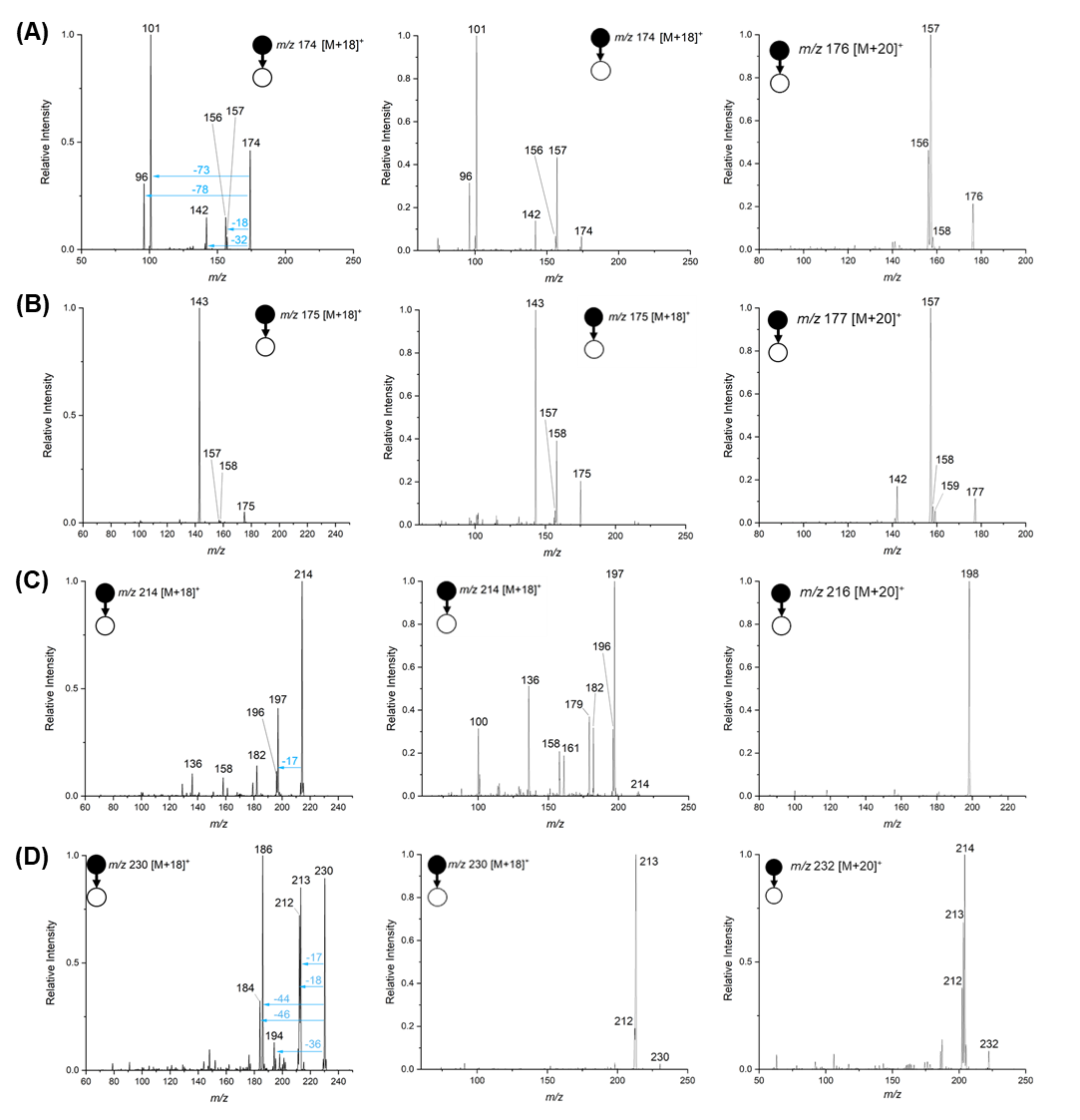
**

**
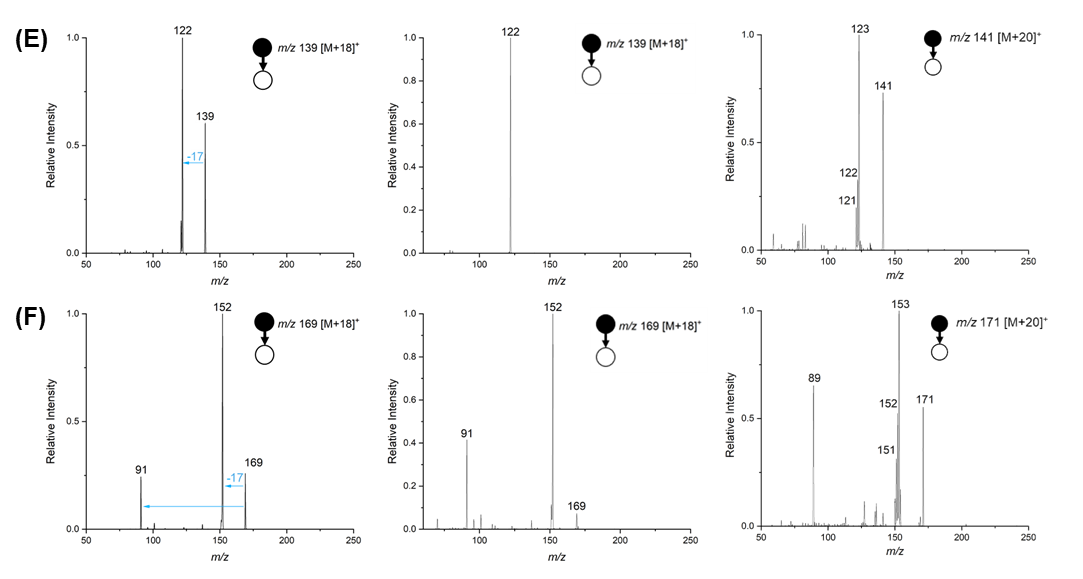
**

**Figure S12.** DESI-MS/MS spectra of the [M+18]^+^˙ corresponding to the six monofunctional model compounds (viz. sulfone (**A**), sulfonamide (**B**), ketone (**C**), ester (**D**), amide (**E**), and carbamate (**F**)), acquired using different DESI solvents: methanol with 0.1% formic acid (***left***), methanol (***center***), and methanol-*d*_4_ (***right***). Note that in the latter case the MS/MS spectra correspond to the precursor ions [M+20]^+^˙ which correspond to the compound adducts with D_2_O^+^˙.

**
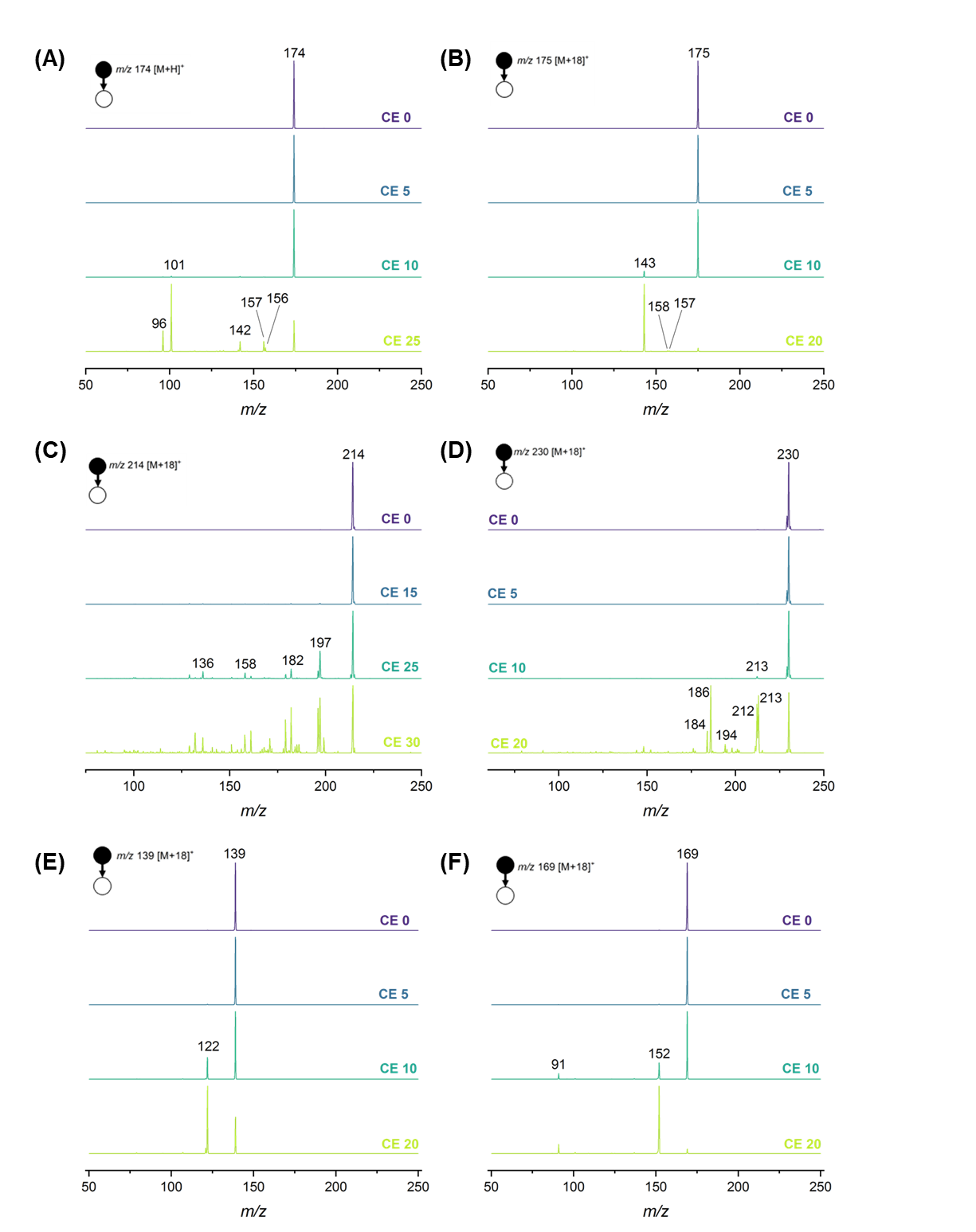
**

**Figure S13.** DESI-MS/MS spectra of the [M+18]^+^˙ ions corresponding to the six monofunctional model compounds (viz. sulfone (**A**), sulfonamide (**B**), ketone (**C**), ester (**D**), amide (**E**), and carbamate (**F**)) acquired under different collision energies (CE), as denoted. All CEs are arbitrary normalized units. Methanol with 0.1% formic acid was used as DESI solvent in all cases.

**
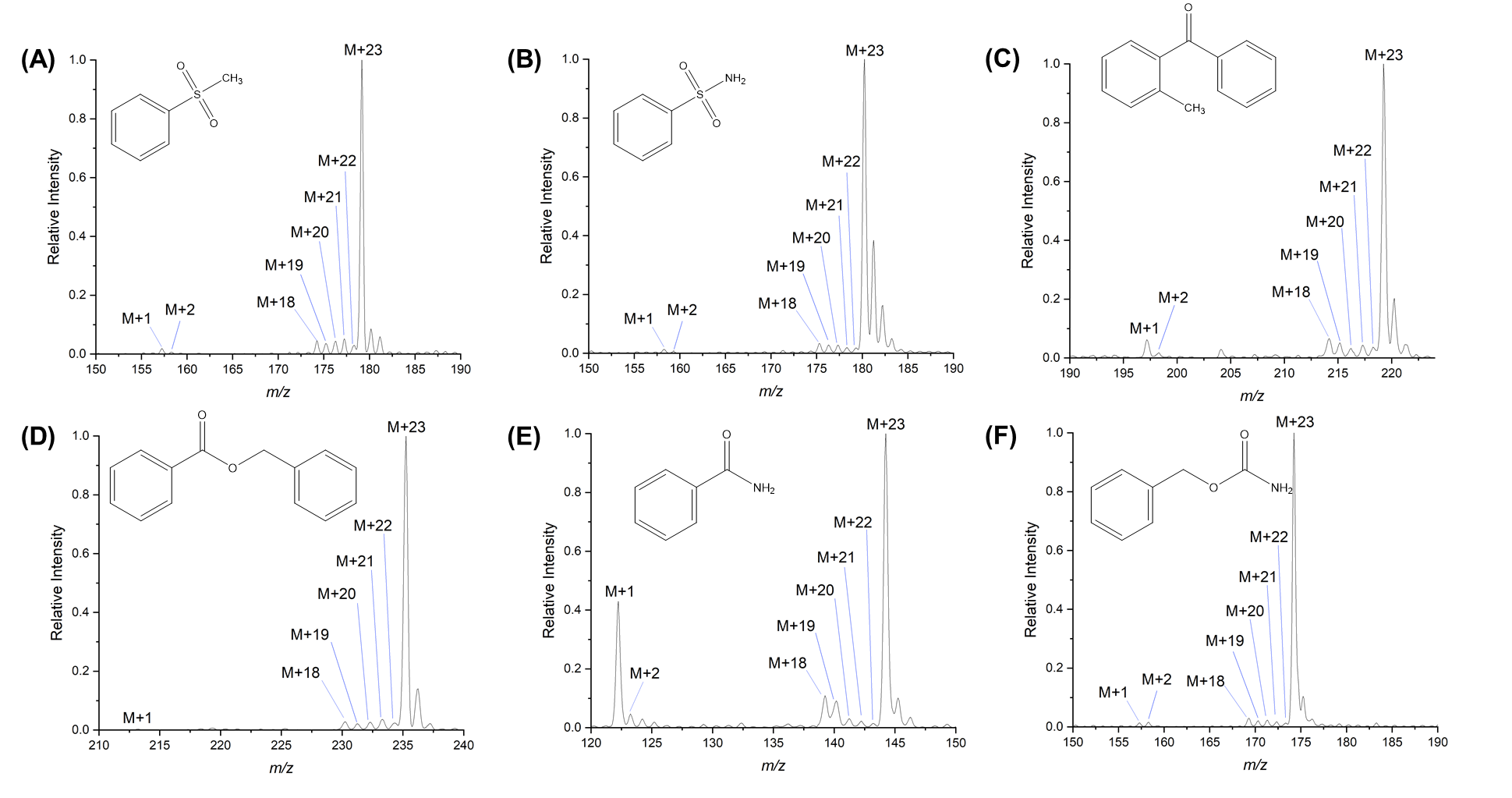
**

**Figure S14.** Mass spectra of six monofunctional compounds in DESI-generated microdroplets using methanol-*d*_4_ as DESI spray solvent. Sulfone (**A**), sulfonamide (**B**), ketone (**C**), ester (**D**), amide (**E**), and carbamate (**F**) functionalities were explored.


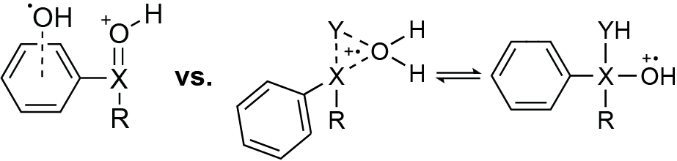


**Figure S15.** Proposed isomer structure (left) for the [M+18]^+^˙ species formed via addition of hydroxyl radical followed by protonation. For the sake of comparison, the proposed structure (two forms, right) for the adduct of water radical cation is also shown.

## Supplementary Tables

**Table S1.** Functional groups identified in the complete compound library. Functional groups are represented by their corresponding SMILES strings.

| **Index** | **Functional group SMILES** | **Index** | **Functional group SMILES** |
| --- | --- | --- | --- |
| 1 | Br | 104 | NC1=NN(C=O)CS1 |
| 2 | C#C | 105 | NC1=NNC2=CNNN21 |
| 3 | C#N | 106 | NC1=NS(=O)(=O)C=C1 |
| 4 | C1CO1 | 107 | NC=C(C=O)C(N)=O |
| 5 | C=C | 108 | NC=C(C=O)C=O |
| 6 | C=C(C(=O)O)C(=O)O | 109 | NC=CC(=O)O |
| 7 | C=C(N)C=O | 110 | NC=CC=O |
| 8 | C=C(NC(=N)O)C(=O)O | 111 | NC=NO |
| 9 | C=C(S)C(N)=O | 112 | NC=NS(=O)=O |
| 10 | C=C1N=COC1=O | 113 | NC=O |
| 11 | C=C1NC(=O)NC1=O | 114 | NC=S |
| 12 | C=C1NC(=S)NC1=O | 115 | NCN |
| 13 | C=CC#N | 116 | NCNC=O |
| 14 | C=CC(=C)C=C | 117 | NN |
| 15 | C=CC(=O)O | 118 | NN=CNC(N)=S |
| 16 | C=CC(N)=O | 119 | NNC=O |
| 17 | C=CC=C | 120 | NNS(=O)=O |
| 18 | C=CC=O | 121 | NO |
| 19 | C=CCl | 122 | NS(=O)=O |
| 20 | C=CN | 123 | NS(N)(=O)=O |
| 21 | C=CN=CN | 124 | NS=O |
| 22 | C=CNC(=S)S | 125 | Nn |
| 23 | C=CNC=O | 126 | O |
| 24 | C=COC(N)=CC#N | 127 | O=C(O)C=CNCO |
| 25 | C=CS(N)(=O)=O | 128 | O=C(O)NC(O)O |
| 26 | C=Cn | 129 | O=C(O)NS(=O)=O |
| 27 | C=N | 130 | O=C(O)O |
| 28 | C=NC(NC=O)C(N)=O | 131 | O=C1NC(=O)C(=O)N1 |
| 29 | C=NN | 132 | O=C1NC(=O)C(S)N1 |
| 30 | C=NN(C=O)CO | 133 | O=C1NC(O)C(O)N1 |
| 31 | C=NNC=CC=O | 134 | O=C1NCNN1 |
| 32 | C=NNC=O | 135 | O=CC=CNC=CC(=O)O |
| 33 | C=NNCO | 136 | O=CC=CNC=CC=O |
| 34 | C=NO | 137 | O=CC=CNC=O |
| 35 | C=O | 138 | O=CC=CO |
| 36 | Cl | 139 | O=CC=COC=CC=O |
| 37 | F | 140 | O=CC=COCO |
| 38 | I | 141 | O=CN=C([O-])NC=O |
| 39 | N | 142 | O=CN=C1NN=C(NC=O)N1 |
| 40 | N#CC(=CN)S(=O)=O | 143 | O=CNC(=O)NC=O |
| 41 | N#CC1=CN=C(S)N2NCN=C12 | 144 | O=CNC(=O)NCS |
| 42 | N#CC=C(N)O | 145 | O=CNC(=O)O |
| 43 | N#CC=C(N)OC=CC=O | 146 | O=CNC(=S)NC=O |
| 44 | N#CC=C(S)NC=O | 147 | O=CNC(O)OC=O |
| 45 | N#CC=C(S)NCO | 148 | O=CNC(S)C(=O)O |
| 46 | N#CC=O | 149 | O=CNC=C(S)C(=O)O |
| 47 | N=C(N)N | 150 | O=CNC=CC(=O)O |
| 48 | N=C(N)NC(N)=S | 151 | O=CNC=O |
| 49 | N=C(N)NC=O | 152 | O=CNCNC=O |
| 50 | N=C(N)S | 153 | O=CNCNS(=O)=O |
| 51 | N=C(NC=O)NC=O | 154 | O=CNCO |
| 52 | N=C(O)C(S)NC=O | 155 | O=CNCS |
| 53 | N=C(S)NC(N)=O | 156 | O=CNCS(=O)=O |
| 54 | N=C(S)NC=O | 157 | O=CNO |
| 55 | N=C1NC=CS1 | 158 | O=CNS(=O)=O |
| 56 | N=CN | 159 | O=CO |
| 57 | N=CN(C=O)C(N)=S | 160 | O=COCO |
| 58 | N=CNC=O | 161 | O=C[N-]C(=S)NC=O |
| 59 | N=CO | 162 | O=C[O-] |
| 60 | N=NN | 163 | O=P(O)O |
| 61 | NC(=O)C(=CO)S(N)(=O)=O | 164 | O=S |
| 62 | NC(=O)C(=O)O | 165 | O=S(=O)CS(=O)=O |
| 63 | NC(=O)C(=O)[O-] | 166 | O=S(=O)N=CNCNS(=O)=O |
| 64 | NC(=O)C(N)=O | 167 | O=S(=O)NCO |
| 65 | NC(=O)C(N)=S | 168 | O=S(=O)NCS |
| 66 | NC(=O)C(N)N | 169 | O=S(=O)NO |
| 67 | NC(=O)C(N)NS(=O)=O | 170 | O=S(=O)NS(=O)=O |
| 68 | NC(=O)C(O)=CC=O | 171 | O=S(=O)O |
| 69 | NC(=O)C(O)=CS(=O)=O | 172 | O=S(=O)[O-] |
| 70 | NC(=O)C(S)NC=O | 173 | O=S1(=O)N=CNCN1 |
| 71 | NC(=O)C=C(S)C(N)=O | 174 | O=S=NS(=O)=O |
| 72 | NC(=O)C=C1NC(=S)NC1=O | 175 | O=S=O |
| 73 | NC(=O)C=CC(=O)O | 176 | O=[N+][O-] |
| 74 | NC(=O)C=CC(N)=O | 177 | OCO |
| 75 | NC(=O)C=CNC(N)=O | 178 | OCOCO |
| 76 | NC(=O)C=CNC(N)=S | 179 | OCS |
| 77 | NC(=O)C=CNC=CC=O | 180 | S |
| 78 | NC(=O)NC(O)C(N)=O | 181 | SCS |
| 79 | NC(=O)NC1=NN(C=O)CS1 | 182 | [N+] |
| 80 | NC(=O)NC=CC(=O)O | 183 | [NH+] |
| 81 | NC(=O)NC=O | 184 | [NH2+] |
| 82 | NC(=O)NCO | 185 | [nH] |
| 83 | NC(=O)NCOC(N)=O | 186 | n |
| 84 | NC(=O)NO | 187 | nN |
| 85 | NC(=O)NS(=O)=O | 188 | nO |
| 86 | NC(=O)O | 189 | nS(=O)=O |
| 87 | NC(=S)NC=CC(=O)O | 190 | nS(N)(=O)=O |
| 88 | NC(=S)NC=CC=O | 191 | n[nH] |
| 89 | NC(=S)NC=O | 192 | nn |
| 90 | NC(=S)NCNC=O | 193 | nn[nH]n |
| 91 | NC(=S)NCO | 194 | nnn |
| 92 | NC(=S)NCS | 195 | nnnO |
| 93 | NC(=S)NNC(N)=S | 196 | nnn[nH] |
| 94 | NC(N)=CC=O | 197 | nnnn |
| 95 | NC(N)=NC=O | 198 | nnnnn |
| 96 | NC(N)=NS(=O)=O | 199 | nns |
| 97 | NC(N)=O | 200 | no |
| 98 | NC(N)=S | 201 | non |
| 99 | NC([O-])=NS(=O)=O | 202 | ns |
| 100 | NC1=NC(C(=O)O)N=CO1 | 203 | nsn |
| 101 | NC1=NCN=CO1 | 204 | o |
| 102 | NC1=NCNC(N)=N1 | 205 | s |
| 103 | NC1=NCNCN1 |  |  |

**Table S2.** Proportion of compounds with a particular functional group (identified by the indexes in Table S1) within the set of molecules that provided a hit for the [M+18]^+^ species and within the whole compound library.

| **Index** | **Proportion with [M+18]^+^** | **Proportion of the total** | **Index** | **Proportion with [M+18]^+^** | **Proportion of the total** |
| --- | --- | --- | --- | --- | --- |
| 1 | 1.7E-02 | 2.0E-02 | 104 | 0.0E+00 | 4.8E-05 |
| 2 | 1.4E-03 | 2.5E-03 | 105 | 0.0E+00 | 9.6E-05 |
| 3 | 6.1E-02 | 4.0E-02 | 106 | 1.1E-03 | 1.0E-03 |
| 4 | 5.4E-04 | 9.6E-05 | 107 | 0.0E+00 | 4.8E-05 |
| 5 | 2.1E-02 | 2.8E-02 | 108 | 0.0E+00 | 1.4E-04 |
| 6 | 0.0E+00 | 4.8E-05 | 109 | 0.0E+00 | 2.4E-04 |
| 7 | 0.0E+00 | 1.4E-04 | 110 | 0.0E+00 | 1.1E-03 |
| 8 | 0.0E+00 | 4.8E-05 | 111 | 0.0E+00 | 1.9E-04 |
| 9 | 0.0E+00 | 4.3E-04 | 112 | 5.4E-03 | 2.6E-03 |
| 10 | 0.0E+00 | 1.9E-04 | 113 | 6.8E-01 | 6.2E-01 |
| 11 | 0.0E+00 | 3.4E-04 | 114 | 0.0E+00 | 3.1E-03 |
| 12 | 2.7E-04 | 3.8E-04 | 115 | 0.0E+00 | 9.6E-04 |
| 13 | 0.0E+00 | 4.8E-05 | 116 | 0.0E+00 | 9.6E-04 |
| 14 | 0.0E+00 | 4.8E-05 | 117 | 0.0E+00 | 9.6E-05 |
| 15 | 4.6E-03 | 1.9E-03 | 118 | 0.0E+00 | 4.8E-05 |
| 16 | 0.0E+00 | 1.9E-04 | 119 | 0.0E+00 | 4.8E-05 |
| 17 | 0.0E+00 | 9.6E-05 | 120 | 0.0E+00 | 4.8E-05 |
| 18 | 1.4E-03 | 8.7E-04 | 121 | 0.0E+00 | 9.6E-05 |
| 19 | 5.4E-04 | 4.8E-04 | 122 | 5.4E-01 | 2.5E-01 |
| 20 | 0.0E+00 | 9.6E-05 | 123 | 1.1E-03 | 8.7E-04 |
| 21 | 0.0E+00 | 1.9E-04 | 124 | 0.0E+00 | 4.8E-05 |
| 22 | 0.0E+00 | 4.8E-05 | 125 | 0.0E+00 | 4.8E-05 |
| 23 | 0.0E+00 | 1.4E-04 | 126 | 4.4E-01 | 4.9E-01 |
| 24 | 0.0E+00 | 4.8E-05 | 127 | 0.0E+00 | 9.6E-05 |
| 25 | 2.7E-04 | 9.6E-05 | 128 | 0.0E+00 | 4.8E-05 |
| 26 | 0.0E+00 | 1.9E-04 | 129 | 0.0E+00 | 1.4E-04 |
| 27 | 0.0E+00 | 1.1E-03 | 130 | 0.0E+00 | 9.6E-05 |
| 28 | 0.0E+00 | 5.8E-04 | 131 | 2.2E-03 | 5.8E-04 |
| 29 | 0.0E+00 | 2.9E-04 | 132 | 0.0E+00 | 4.8E-05 |
| 30 | 0.0E+00 | 9.6E-05 | 133 | 0.0E+00 | 4.8E-05 |
| 31 | 2.7E-04 | 9.6E-05 | 134 | 0.0E+00 | 4.8E-05 |
| 32 | 8.1E-04 | 1.6E-03 | 135 | 0.0E+00 | 1.0E-03 |
| 33 | 0.0E+00 | 4.8E-05 | 136 | 1.4E-03 | 8.7E-04 |
| 34 | 5.4E-04 | 7.2E-04 | 137 | 0.0E+00 | 2.9E-04 |
| 35 | 8.0E-02 | 5.6E-02 | 138 | 0.0E+00 | 1.4E-04 |
| 36 | 2.2E-01 | 2.0E-01 | 139 | 2.7E-03 | 6.7E-04 |
| 37 | 2.3E-01 | 2.1E-01 | 140 | 0.0E+00 | 4.8E-05 |
| 38 | 2.7E-04 | 7.2E-04 | 141 | 0.0E+00 | 4.8E-05 |
| 39 | 2.6E-02 | 4.2E-01 | 142 | 0.0E+00 | 4.8E-05 |
| 40 | 0.0E+00 | 1.4E-04 | 143 | 0.0E+00 | 9.6E-05 |
| 41 | 0.0E+00 | 4.8E-05 | 144 | 0.0E+00 | 4.8E-05 |
| 42 | 2.7E-04 | 1.9E-04 | 145 | 2.7E-04 | 4.3E-04 |
| 43 | 5.4E-04 | 1.9E-04 | 146 | 0.0E+00 | 9.6E-05 |
| 44 | 1.1E-03 | 1.9E-04 | 147 | 2.7E-04 | 4.8E-05 |
| 45 | 5.4E-04 | 9.6E-05 | 148 | 0.0E+00 | 4.8E-05 |
| 46 | 0.0E+00 | 4.8E-05 | 149 | 0.0E+00 | 4.8E-05 |
| 47 | 0.0E+00 | 3.4E-04 | 150 | 8.1E-04 | 1.9E-04 |
| 48 | 0.0E+00 | 4.8E-05 | 151 | 1.8E-02 | 1.4E-02 |
| 49 | 0.0E+00 | 4.8E-05 | 152 | 2.7E-04 | 9.6E-05 |
| 50 | 0.0E+00 | 1.5E-03 | 153 | 0.0E+00 | 1.4E-04 |
| 51 | 0.0E+00 | 4.8E-05 | 154 | 8.1E-04 | 8.2E-04 |
| 52 | 2.7E-04 | 4.8E-05 | 155 | 5.4E-04 | 4.8E-04 |
| 53 | 0.0E+00 | 9.6E-05 | 156 | 0.0E+00 | 4.8E-05 |
| 54 | 0.0E+00 | 1.2E-03 | 157 | 8.1E-04 | 5.3E-04 |
| 55 | 0.0E+00 | 4.8E-05 | 158 | 5.2E-03 | 1.9E-03 |
| 56 | 2.7E-04 | 1.3E-03 | 159 | 2.6E-01 | 1.7E-01 |
| 57 | 0.0E+00 | 4.8E-04 | 160 | 0.0E+00 | 4.8E-05 |
| 58 | 0.0E+00 | 9.6E-05 | 161 | 0.0E+00 | 9.6E-05 |
| 59 | 2.7E-04 | 9.6E-05 | 162 | 8.1E-04 | 4.8E-04 |
| 60 | 0.0E+00 | 4.8E-05 | 163 | 0.0E+00 | 5.3E-04 |
| 61 | 0.0E+00 | 4.8E-05 | 164 | 2.4E-03 | 1.3E-03 |
| 62 | 2.4E-03 | 5.8E-04 | 165 | 0.0E+00 | 4.8E-05 |
| 63 | 0.0E+00 | 4.8E-05 | 166 | 0.0E+00 | 4.8E-05 |
| 64 | 9.5E-03 | 7.1E-03 | 167 | 2.7E-04 | 1.4E-04 |
| 65 | 2.7E-04 | 3.4E-04 | 168 | 5.4E-04 | 3.4E-04 |
| 66 | 0.0E+00 | 1.4E-04 | 169 | 0.0E+00 | 4.8E-05 |
| 67 | 2.7E-04 | 1.4E-04 | 170 | 8.1E-04 | 1.9E-04 |
| 68 | 1.6E-03 | 1.3E-03 | 171 | 0.0E+00 | 2.9E-04 |
| 69 | 2.7E-04 | 9.6E-05 | 172 | 0.0E+00 | 4.8E-05 |
| 70 | 5.4E-04 | 1.9E-04 | 173 | 0.0E+00 | 9.6E-05 |
| 71 | 0.0E+00 | 9.6E-05 | 174 | 1.1E-03 | 1.9E-04 |
| 72 | 0.0E+00 | 4.8E-05 | 175 | 5.1E-02 | 2.4E-02 |
| 73 | 0.0E+00 | 1.4E-04 | 176 | 2.3E-02 | 2.1E-02 |
| 74 | 0.0E+00 | 4.8E-05 | 177 | 1.1E-02 | 2.1E-02 |
| 75 | 0.0E+00 | 2.4E-04 | 178 | 2.7E-04 | 1.4E-04 |
| 76 | 0.0E+00 | 5.3E-04 | 179 | 2.7E-04 | 4.8E-05 |
| 77 | 0.0E+00 | 3.4E-04 | 180 | 6.1E-02 | 7.4E-02 |
| 78 | 0.0E+00 | 9.6E-05 | 181 | 2.7E-04 | 9.6E-05 |
| 79 | 0.0E+00 | 4.8E-05 | 182 | 0.0E+00 | 1.2E-03 |
| 80 | 0.0E+00 | 1.6E-03 | 183 | 0.0E+00 | 1.4E-04 |
| 81 | 2.4E-02 | 1.5E-02 | 184 | 0.0E+00 | 4.8E-05 |
| 82 | 0.0E+00 | 6.7E-04 | 185 | 1.8E-02 | 4.6E-02 |
| 83 | 0.0E+00 | 4.8E-05 | 186 | 6.0E-02 | 2.7E-01 |
| 84 | 0.0E+00 | 1.4E-04 | 187 | 0.0E+00 | 4.8E-05 |
| 85 | 2.4E-03 | 9.1E-04 | 188 | 8.1E-04 | 1.4E-04 |
| 86 | 5.9E-02 | 3.2E-02 | 189 | 0.0E+00 | 3.4E-04 |
| 87 | 0.0E+00 | 9.1E-04 | 190 | 0.0E+00 | 4.8E-05 |
| 88 | 0.0E+00 | 4.8E-05 | 191 | 1.9E-03 | 3.3E-03 |
| 89 | 1.6E-03 | 2.6E-03 | 192 | 1.4E-02 | 6.1E-02 |
| 90 | 0.0E+00 | 4.8E-05 | 193 | 0.0E+00 | 3.4E-04 |
| 91 | 0.0E+00 | 9.6E-05 | 194 | 1.1E-03 | 2.2E-03 |
| 92 | 0.0E+00 | 4.8E-05 | 195 | 0.0E+00 | 4.8E-05 |
| 93 | 0.0E+00 | 4.8E-05 | 196 | 8.1E-04 | 3.8E-04 |
| 94 | 0.0E+00 | 4.8E-05 | 197 | 6.8E-03 | 7.7E-03 |
| 95 | 0.0E+00 | 9.6E-05 | 198 | 0.0E+00 | 1.4E-04 |
| 96 | 0.0E+00 | 9.6E-05 | 199 | 8.1E-04 | 2.9E-04 |
| 97 | 3.3E-02 | 5.4E-02 | 200 | 1.6E-02 | 1.9E-02 |
| 98 | 2.7E-03 | 3.7E-02 | 201 | 5.4E-04 | 7.2E-04 |
| 99 | 0.0E+00 | 4.8E-05 | 202 | 1.1E-03 | 3.1E-03 |
| 100 | 0.0E+00 | 4.8E-05 | 203 | 2.2E-03 | 2.6E-03 |
| 101 | 0.0E+00 | 9.6E-05 | 204 | 2.1E-02 | 3.0E-02 |
| 102 | 0.0E+00 | 9.6E-05 | 205 | 1.1E-01 | 1.5E-01 |
| 103 | 0.0E+00 | 9.6E-05 |  |  |  |

# References

Aureliano Antunes, C. S., Bietti, M., Ercolani, G., Lanzalunga, O., and Salamone, M. (2005). The effect of ring substitution on the O-neophyl rearrangement of 1,1-diarylalkoxyl radicals. A product and time-resolved kinetic study. *J. Org. Chem.* 70, 3884–3891. doi:10.1021/jo0502448.

Morato, N. M., Le, M. P. T., Holden, D. T., and Graham Cooks, R. (2021). Automated High-Throughput System Combining Small-Scale Synthesis with Bioassays and Reaction Screening. *SLAS Technol.* 26, 555–571. doi:10.1177/24726303211047839.

Nie, H., Wei, Z., Qiu, L., Chen, X., Holden, D. T., and Cooks, R. G. (2020). High-yield gram-scale organic synthesis using accelerated microdroplet/thin film reactions with solvent recycling. *Chem. Sci.* 11, 2356–2361. doi:10.1039/c9sc06265c.

Qiu, L., Psimos, M. D., and Cooks, R. G. (2022). Spontaneous Oxidation of Aromatic Sulfones to Sulfonic Acids in Microdroplets. *J. Am. Soc. Mass Spectrom.*, jasms.2c00029. doi:10.1021/jasms.2c00029.

Qiu, L., Wei, Z., Nie, H., and Cooks, R. G. (2021). Reaction Acceleration Promoted by Partial Solvation at the Gas/Solution Interface. *Chempluschem* 86, 1362–1365. doi:10.1002/cplu.202100373.

Sobreira, T. J. P., Avramova, L., Szilagyi, B., Logsdon, D. L., Loren, B. P., Jaman, Z., et al. (2020). High-throughput screening of organic reactions in microdroplets using desorption electrospray ionization mass spectrometry (DESI-MS): hardware and software implementation. *Anal. Methods* 12, 3654–3669. doi:10.1039/D0AY00072H.
